# Leveraging conformal prediction to annotate enzyme function space with limited false positives

**DOI:** 10.1371/journal.pcbi.1012135

**Published:** 2024-05-29

**Authors:** Kerr Ding, Jiaqi Luo, Yunan Luo

**Affiliations:** School of Computational Science and Engineering, Georgia Institute of Technology, Atlanta, Georgia, United States of America; University of Virginia, UNITED STATES

## Abstract

Machine learning (ML) is increasingly being used to guide biological discovery in biomedicine such as prioritizing promising small molecules in drug discovery. In those applications, ML models are used to predict the properties of biological systems, and researchers use these predictions to prioritize candidates as new biological hypotheses for downstream experimental validations. However, when applied to unseen situations, these models can be overconfident and produce a large number of false positives. One solution to address this issue is to quantify the model’s prediction uncertainty and provide a set of hypotheses with a controlled false discovery rate (FDR) pre-specified by researchers. We propose CPEC, an ML framework for FDR-controlled biological discovery. We demonstrate its effectiveness using enzyme function annotation as a case study, simulating the discovery process of identifying the functions of less-characterized enzymes. CPEC integrates a deep learning model with a statistical tool known as conformal prediction, providing accurate and FDR-controlled function predictions for a given protein enzyme. Conformal prediction provides rigorous statistical guarantees to the predictive model and ensures that the expected FDR will not exceed a user-specified level with high probability. Evaluation experiments show that CPEC achieves reliable FDR control, better or comparable prediction performance at a lower FDR than existing methods, and accurate predictions for enzymes under-represented in the training data. We expect CPEC to be a useful tool for biological discovery applications where a high yield rate in validation experiments is desired but the experimental budget is limited.

## Author summary

Machine learning (ML) models are increasingly being applied as predictors to generate biological hypotheses and guide biological discovery. However, when applied to unseen situations, ML models can be overconfident and make enormous false positive predictions, resulting in the challenges for researchers to trade-off between high yield rates and limited budgets. One solution is to quantify the model’s prediction uncertainty and generate predictions at a controlled false discovery rate (FDR) pre-specified by researchers. Here, we introduce CPEC, an ML framework designed for FDR-controlled biological discovery. Using enzyme function prediction as a case study, we simulate the process of function discovery for less-characterized enzymes. Leveraging a statistical framework, conformal prediction, CPEC provides rigorous statistical guarantees that the FDR of the model predictions will not surpass a user-specified level with high probability. Our results suggested that CPEC achieved reliable FDR control for enzymes under-represented in the training data. In the broader context of biological discovery applications, CPEC can be applied to generate high-confidence hypotheses and guide researchers to allocate experimental resources to the validation of hypotheses that are more likely to succeed.

## Introduction

Machine learning (ML) algorithms have proven to be transformative tools for generating biological hypotheses and uncovering knowledge from large datasets [1, 2]. Applications include designing function-enhanced proteins [3, 4], searching for novel drug molecules [5], and optimizing human antibodies against new viral variants [6]. These discoveries often involve a combination of computation and experimentation, where ML-based predictive models generate biological hypotheses and wet-lab experiments are then used to validate them. This approach is beneficial as it greatly reduces the search space and eliminates candidates that are unlikely to be successful, thus saving time and resources in the discovery process. For example, in drug discovery, ML has become a popular strategy for virtual screening of molecule libraries, where researchers use ML models to predict the properties of molecules, such as binding affinity to a target, and identify the most promising candidates for downstream experimental validation and lead optimization [7].

To gain new insights into biological systems or make novel discoveries (e.g., designing new drugs), ML algorithms are often used to make predictions for previously unseen data samples. For example, to support the design of new vaccines or therapeutics for COVID-19, ML algorithms need to predict the potential for immune escape of future variants that are composed of mutations that have not yet been seen. Similarly, in drug screening, ML algorithms should be able to predict molecules that are structurally different from those in the training data, which helps scientists avoid re-discovering existing drugs. However, making predictions for samples that are under-represented in the training data is a challenging task in ML. While human experts can assess the success likelihood of generated hypotheses based on their domain knowledge or intuition, this ability is not naturally developed by an ML model and, as a result, the model could be susceptible to pathological failure and only provide overconfident or unreliable predictions. This can have critical implications in ML-assisted biological discovery, as unreliable ML predictions can guide experimental efforts in the wrong direction, wasting resources on validating false positives.

In this work, we aim to develop ML models that can generate hypotheses with limited false positives, providing confident and accurate predictions that can potentially help improve the yield rate in downstream validation experiments. Specifically, we use the function annotation problem of protein enzymes as an example to demonstrate our method. The underlying computational problem of function annotation is a multi-class, multi-label classification problem as a protein can have multiple functions. In computational protein function annotation, a model typically predicts a set of functions that the query protein may potentially have. The set of predicted functions, if validated by experiments, can be incorporated into existing databases to augment our knowledge of the protein function space. There is often a trade-off regarding the size of the prediction set: researchers prefer a set with a small size, containing a handful of very confident predictions, as it is not desirable to spend resources on too many hypotheses that ultimately turn out to be false positives; on the other hand, researchers may be willing to increase the budget to validate a larger set of predictions in order to improve the chance of discovering novel functions for under-studied proteins.

The above tradeoff is often captured by different notions of prediction score cutoff, which decides whether to assign a particular function label to a protein, in existing computational methods for function annotation. For example, when annotating protein functions using sequence-similarity-based tools such as BLAST [8], a cutoff of the BLAST E-value can be used to determine the significance of sequence match. However, the choice of E-value cutoff is often based on the user’s intuition and good cutoff values on a dataset may not generalize to another dataset. Recent ML methods for enzyme function annotation typically first predict the probability that the input protein has a particular function and annotate the protein with this function if the predicted probability is greater than 0.5 [9–11]. However, using an arbitrary cutoff such as 0.5 is problematic as the predicted probabilities do not always translate to the confidence of the ML model, especially when the model is not well-calibrated (e.g., a predicted function with probability 0.95 may still be an unreliable prediction if the model is overconfident and produces very high probability scores most of the time). Recently, Hie et al. [12] developed a framework that used the Gaussian process to estimate the confidence or uncertainty in the ML model’s predictions. While the framework was shown to be effective to guide biological discovery, it is unclear how the estimated uncertainty is related to the final false discovery rate (FDR) in experimental validation and how to set a cutoff on the uncertainty scores to achieve a desired FDR. Consequently, it is challenging to provide FDR estimates before the experimental validation, and FDR typically can only be assessed post-validation.

Here, we propose an ML method, called CPEC (Conformal Prediction of EC number), to achieve FDR-controlled enzyme function prediction by leveraging a statistical framework known as conformal prediction (CP) [13]. CPEC receives the sequence or structure of an enzyme as input and predicts a set of functions (EC numbers) that the enzyme potentially has. The unique strength of CPEC is that the averaged per-protein FDR (i.e., the number of incorrect predictions divided by the prediction set size for a protein) can be controlled by a user-specified hyper-parameter *α*. The CP framework theoretically guarantees that the FDR of our per-protein predictions is no larger than *α* with a very high probability. This equips researchers with foresight, offering success rate estimates even before experimental validation. In an ML-guided workflow of protein function discovery, researchers can specify the desired FDR level *α* based on the experiment budget or expectations. For example, setting *α* to a smaller value when only the most confident predictions are needed or the test budget is limited, or setting to a larger value when the goal is to discover novel functions and a slightly higher FDR and budget are acceptable. The base ML model of CPEC is PenLight2, an improved version of the deep learning model PenLight [14] for the multi-class multi-label protein function annotation problem, which uses a graph neural network to integrate 3D protein structure data and protein language model embeddings to learn structure-aware representations for function prediction. Benchmarked on a carefully curated dataset, we first found that CPEC outperformed existing deep learning methods for enzyme function prediction. We also demonstrated that CPEC provides rigorous guarantees of FDR and allows users to trade-off between precision and recall in the predictions by tuning the desired maximum value *α* of FDR. Additionally, we showed that CPEC consistently provides FDR-controlled predictions for proteins with different sequence identities to the training set, suggesting its robustness even in regimes beyond its training data distribution. Moreover, based on CPEC, we proposed a cascade model that can better balance the resolution and coverage for EC number prediction.

## Materials and methods

### Problem formulation

**Fig 1:**
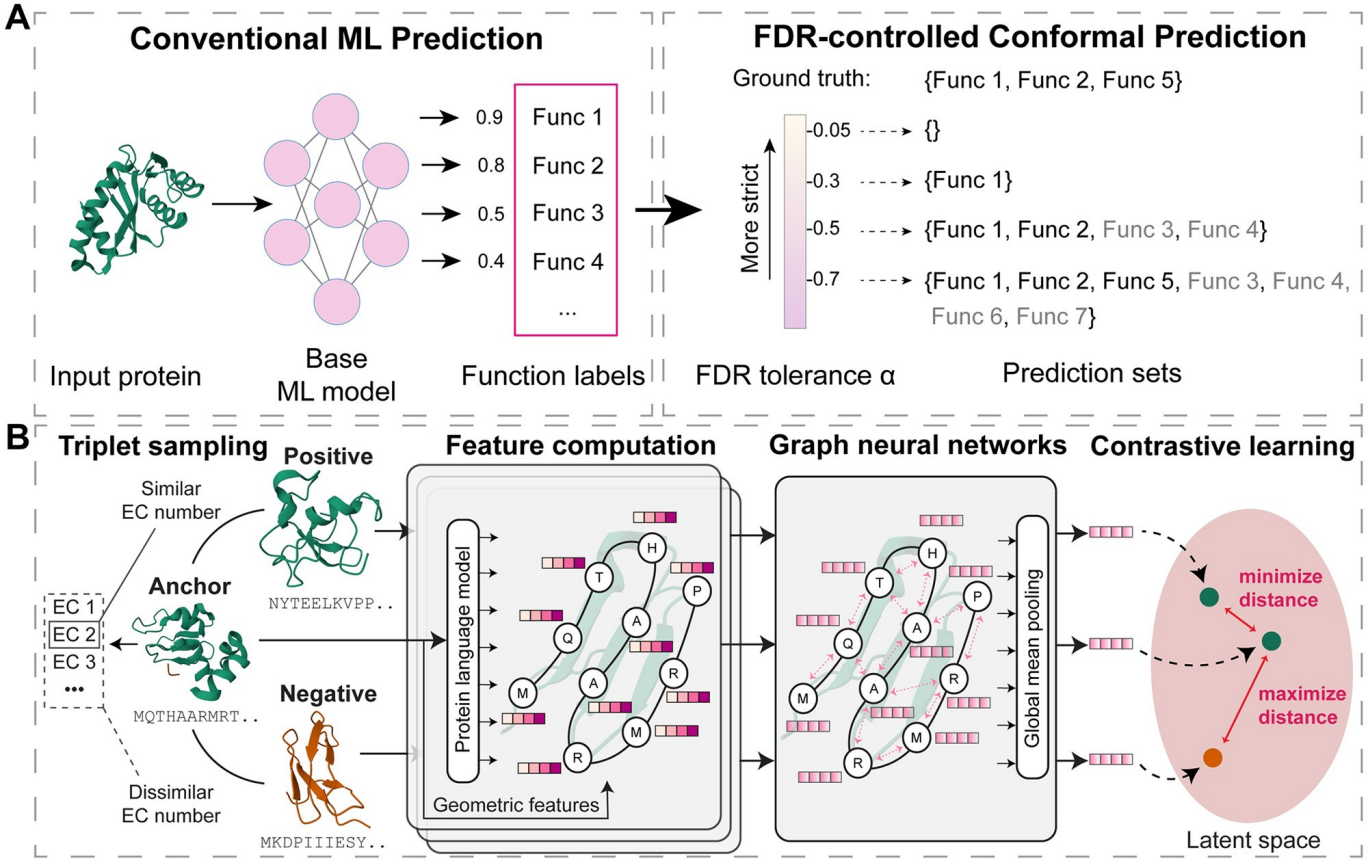
Schematic overview of CPEC. (A) CPEC is a machine learning (ML) framework that leverages conformal prediction to control the false discovery rate (FDR) while performing enzyme function predictions. Compared to conventional ML predictions, CPEC allows users to select the desired FDR tolerance *α* and generates corresponding FDR-controlled prediction sets. Enabled by conformal prediction, CPEC provides a rigorous statistical guarantee such that the FDR of its predictions will not exceed the FDR tolerance *α* set by the users. The FDR tolerance *α* offers flexibilities in ML-guided biological discovery: when *α* is small, CPEC only produces hypotheses for which it has the most confidence; a larger *α* value would allow CPEC to afford a higher FDR, and CPEC thus can predict a set with more function labels to improve the true positive rate. Abbreviation: Func: function. Incorrect predictions in prediction sets are colored gray. (B) We developed a deep learning model, PenLight2, as the base model of the CPEC framework. The model is a graph neural network that receives the three-dimensional structure and the sequence of a protein as input and generates a function-aware representation for the protein. It employs a contrastive learning scheme to learn a vector representation for proteins, such that the representations of functionally similar proteins in the latent space are pulled together while dissimilar proteins are pushed apart.

Protein function prediction, such as Gene Ontology (GO) term [15] prediction [16] and EC number [17] prediction, can be formulated as a multi-class, multi-label classification problem, where each protein can have multiple ground-truth labels. For the set of all possible proteins X and the ground truth label set Y={1,…,K}, an ML model $f:X\mapsto {\left[0,1\right]}^{K}$ for protein function prediction predicts the probability of the input protein X∈X having each function label *k* ∈ {1, …, *K*}. In application, a decision threshold parameter λ is often needed to generate the final prediction set $C_{\lambda }\left(X\right)=\left\{k\in Y:f_{k}\left(X\right)\geq \lambda \right\}$. Instead of choosing an arbitrary constant cutoff (e.g., λ = 0.5), *conformal prediction* (CP), also known as *conformal risk control*, uses a small set of calibration data to select a valid parameter λ that would satisfy rigorous statistical guarantees for model mistakes on test data, based on the user-defined risk tolerance. As false discovery rate (FDR) reflects how much proportion of the experimental validation of ML prediction results would be unsuccessful, it is directly related to the gains out of the wet lab experiments as opposed to the experimental costs, which is a tradeoff researchers often need to confront with. Therefore, in our work, we define FDR as the model mistakes and focus on controlling the FDR of ML predictions. We propose an ML framework, CPEC, which leverages the conformal prediction framework to achieve FDR-controlled enzyme functions prediction, and we developed a deep learning model PenLight2 as the base ML model of the CPEC framework (Fig 1).

### Conformal risk control

#### Overview of conformal risk control

Conformal risk control, the generalization of conformal prediction [18–20], is a paradigm applicable to general ML models for prediction, which generates prediction sets with rigorous statistical guarantees for a user-defined level of model mistakes [21]. Conformal risk control algorithms begin with a trained ML model $\hat{f}$ and its decision threshold parameter λ, as previously defined. Note that conformal risk control does not require additional training or fine-tuning of the existing trained model. Through the calibration step on a small calibration set $D_{cal}={\left\{\left(X_{i}^{c},Y_{i}^{c}\right)\right\}}_{i=1}^{n_{c}}$ that the model $\hat{f}$ has not encountered with during training, the algorithms could determine a suitable decision threshold λ, which could control the risks on the test set $D_{test}={\left\{\left(X_{i}^{t},Y_{i}^{t}\right)\right\}}_{i=1}^{n_{t}}$ as the user’s request. In terms of the definition of the model mistakes, existing conformal risk control algorithms allow users to select the mistake type that they would like to focus on from numerous options (e.g., miscoverage [19], false negative rate (FNR) [21], and FDR [22]).

#### Conformal risk control guarantee for FDR control

Regarding different model mistake types, the conformal risk control guarantees also take distinct forms. For example, the *conformal coverage guarantee* [23] and the *conformal risk control guarantee* [21] target risk functions that are monotonically non-increasing with respect to the model parameter λ, which is explained in details in S1 Text. As FDR does not strictly hold for this monotonic requirement, we have to apply a more general definition of risk guarantee [18, 22] which enables the control of general risks instead of certain monotonic risks. For any user-defined risk function *l*(⋅, ⋅), this generalized guarantee takes the below form:

$$\begin{array}{c} P(R(\lambda )\leq \alpha )\geq 1-\delta , \end{array}$$ (1)

where $R\left(\lambda \right)=E_{D_{test}}[l(C_{\lambda }\left(X\right),Y)]$ is the notion of the risk, *α* is the user-defined risk tolerance, and failure rate *δ* refers to the upper bound of probability of *R*(λ) not falling below *α*. In CPEC, if not otherwise specified, we always use *δ* = 0.1 following the convention of previous studies [13] and fixed *δ* throughout the experiments. The intuition of Eq 1 is that the risk of the prediction sets on test samples will fall under the risk tolerance *α* with a probability of at least 1 − *δ*. The risk function of FDR is defined as below [22]:

$$\begin{array}{c} l_{\text{FDR}}\left(C_{\lambda }\left(X\right),Y\right)=1-\frac{|C_{\lambda }\left(X\right)\cap Y|}{|C_{\lambda }\left(X\right)|}, \end{array}$$ (2)

where the output of *l*_FDR_ is defined as 0 if *C*_λ_(*X*) is an empty set. Consequently, the objective of controlling the FDR of the predictions within the CPEC framework can be formulated as:

$$\begin{array}{c} P(E_{D_{test}}[l_{\text{FDR}}\left(C_{\lambda }\left(X\right),Y\right)]\leq \alpha )\geq 1-\delta . \end{array}$$ (3)

#### Calibration algorithm for FDR control

Given the FDR control guarantee, the natural follow-up question would be how to find a valid parameter λ that can control the risk through the calibration step on calibration data. The *Learn then Test* (LTT) algorithm [22], which formulated the selection of λ as a multiple hypotheses testing problem, has been proposed to solve this question. CPEC adopts the LTT algorithm established upon the data distribution assumption that all feature-response pairs (*X*, *Y*) from the calibration set and the test set are independent and identically distributed (*i.i.d.*).

For the candidate model parameter set Λ = {λ_1_, …, λ_*N*_} where λ_1_ < λ_2_ < ⋯ < λ_*N*_, the LTT algorithm associate each λ_*i*_ with a null hypothesis $H_{i}:R\left(\lambda_{i}\right)>\alpha$. The rejection of the null hypothesis $H_{j}$ would mean that λ_*i*_ can control the user-specified risk at the defined level. As FDR is nearly a monotonically decreasing function of λ, testing for larger λ is more likely to reject the hypothesis than testing for smaller λ. Following Angelopoulos et al. [22], we adopted the *fixed sequence testing* algorithm [24] for FDR control, which tests the multiple hypotheses sequentially from λ_*N*_ to λ_1_, stops upon the first acceptance of the null hypothesis, and eventually returns a rejection set $\hat{\Lambda }$. While any $\lambda \in \hat{\Lambda }$ can control the risks, we select $\lambda =\text{min}\hat{\Lambda }$ as the ultimate threshold for making the predictions on test proteins because the smallest λ provides the largest number of successful discoveries and has the least FNR [22].

According to the data distribution assumption that the calibration and test feature-response pairs (*X*, *Y*) are *i.i.d.*, we are able to test the null hypothesis on the calibration set and then apply the derived parameter to the test set. For each null hypothesis $H_{i}$, we calculate a corresponding p-value *p*_*i*_: a *p*_*i*_ no greater than *δ* would imply the disagreement with $H_{i}$ and the successful control of the risk as the user requests. The commonly used p-values include Hoeffding’s inequality-based p-values [25] and Hoeffding-Bentkus inequality-based p-values [26].

In CPEC, we tested a total of *N* = 100 evenly spaced candidate parameters between [0, 1], with λ_1_ = 0 and λ_*N*_ = 1. The calibration algorithm of CPEC for FDR control described above is given in Algorithm 1. We used Hoeffding’s inequality p-values for hypothesis testing, which is defined below:

$$\begin{array}{c} p_{\lambda }^{\text{Hoeffding}}=\text{exp}\left(-2n_{c}{\left(\alpha -\hat{R}\left(\lambda \right)\right)}_{+}^{2}\right), \end{array}$$ (4)

where $\hat{R}\left(\lambda \right)=\frac{1}{n_{c}}\sum_{i=1}^{n_{c}}l\left(C_{\lambda }\left(X_{i}^{c}\right),Y_{i}^{c}\right)$ is the empirical risks on the calibration data, (⋅)_+_ is the ReLU function, and *n*_*c*_ is the size of the calibration set. The proofs of the validity of Hoeffding’s inequality p-values are included in S1 Text.

**Algorithm 1:** CPEC for FDR control

**Input**: FDR tolerance *α* ∈ (0, 1), failure rate *δ*, total number of candidate parameters *N*, candidate parameter set Λ = {λ_1_, …, λ_*N*_}, calibration data $D_{cal}={\left\{(X_{i},Y_{i})\right\}}_{i=1}^{n_{c}}$

/* Calculation of Hoeffding’s inequality p-values {*p*_1_, …, *p*_*N*_}    */

**for** *i* ← 1 to *N* **do**

 $\hat{R}\left(\lambda_{i}\right)\leftarrow 0$;

 **for** *j* ← 1 to *n*_*c*_ **do**

  **if** $|C_{\lambda_{i}}\left(X_{j}\right)|>0$ **then**

   $\hat{R}\left(\lambda_{i}\right)\leftarrow \hat{R}\left(\lambda_{i}\right)+(1-|C_{\lambda_{i}}\left(X_{j}\right)\cap Y_{j}\left|/\right|C_{\lambda_{i}}\left(X_{j}\right)\left|\right)$;

  **end**

 **end**

 $\hat{R}\left(\lambda_{i}\right)\leftarrow \hat{R}\left(\lambda_{i}\right)/n_{c}$;

 $p_{i}\leftarrow \text{exp}\left(-2n_{c}{\left(\alpha -\hat{R}\left(\lambda_{i}\right)\right)}_{+}^{2}\right)$;

**end**

/* Fixed sequence testing for rejection set $\hat{\Lambda }$    */

$\hat{\Lambda }\leftarrow ⌀$ ;

*i* ← *N*;

**while** *p*_*i*_ ≤ *δ* and *i* ≥ 1 **do**

 $\hat{\Lambda }\leftarrow \hat{\Lambda }\cup \left\{\lambda_{i}\right\}$

 *i* ← *i* − 1;

**end**

**Return**: valid parameter $\lambda =\text{min}\;\hat{\Lambda }$

### Protein function prediction

#### EC number prediction dataset

We applied CPEC on the task of Enzyme Commission (EC) numbers [17] prediction to demonstrate its effectiveness. EC number is a widely used four-level classification scheme, which organizes the protein according to their functions of catalyzing biochemical reactions. In specific, a protein would be labeled with an EC number if it catalyzes the type of biochemical reactions represented by that EC number. For each four-digit EC number *a*.*b*.*c*.*d*, the 1st-level *a* is the most general classification level while the 4th-level *d* is the most specific one. We used the dataset that contains EC number-labeled protein sequences and structures, provided by Gligorijević et al. [10]. The protein structures were retrieved from Protein Data Bank (PDB) [27]. Protein chains were then clustered at 95% sequence identity using the BLASTClust function in the BLAST tool [8] and then organized into a non-redundant set which only included one labeled high-resolution protein chain from each cluster. The EC number annotations were collected from SIFTS (structure integration with function, taxonomy, and sequence) [28]. As the 4th-level EC number is the most informative functional label, we only kept proteins that have ground-truth level-4 EC numbers in our experiments. Eventually, the dataset we used has 10, 245 proteins and a train/valid/test ratio of roughly 7: 1: 2. The proteins in the test set have a maximum sequence identity of 95% to the training set. Within the test set, test proteins were further divided into disjoint groups with [0, 30%), [30%, 40%), [40%, 50%), [50%, 70%), and [70%, 95%] sequence identity to the training set. The lower the sequence identity to the training set, the more difficult the test protein would be for ML models to predict its functions. In experiments, we have used the more challenging test data group ([0, 30%)) to evaluate the robustness of our framework.

#### Contrastive learning-based protein function prediction

For protein function prediction tasks, supervised learning has long been a popular choice in the deep learning community. Supervised learning-based methods take protein sequences or structures as input and directly map them into class labels. While the idea is simple and efficient, supervised learning has been suffering from a major drawback: its performance could be severely affected by the class imbalances of the training data, an unfortunately common phenomenon in protein function prediction tasks. For example, in the EC number database, some EC classes contain very few proteins (less than ten), while some other EC classes contain more than a hundred proteins. Those classes with more proteins would dominate the training, thereby suppressing the minority classes and degrading the performance of supervised learning. To overcome this challenge, a new paradigm called contrastive learning has become popular in recent years [29]. Instead of directly outputting class labels, contrastive learning-based models map the training proteins into an embedding space where functionally similar proteins are close to each other and functionally dissimilar pairs are far away. Our previously developed ML methods PenLight and CLEAN [14, 30] have demonstrated the effectiveness of contrastive learning in enzyme function predictions. In each iteration of the contrastive learning process, the PenLight or CLEAN model samples a triplet including an anchor protein *p*_0_, a positive protein *p*_+_, and a negative protein *p*_−_, such the positive protein pairs (*p*_0_, *p*_+_) have similar EC numbers (e.g., under the same subtree in the EC number ontology) while the negative pairs (*p*_0_, *p*_−_) have dissimilar EC numbers. The objective of contrastive learning is to learn low-dimensional embeddings ***x***_0_, ***x***_+_, ***x***_−_ for the protein triplet such that the embedding distance *d*(***x***_0_, ***x***_+_) is minimized while *d*(***x***_0_, ***x***_−_) is maximized (Fig 1B and S1 Text). In the prediction time, the EC number of the training protein with the closest embedding distance to the query protein will be used as the predicted function labels for the query protein.

In this work, we developed PenLight2, an extension of our previous PenLight model [14] for performing multi-label classification of EC numbers. Similar to PenLight, PenLight2 is a structure-based contrastive learning framework that integrates protein sequence and structure data for predicting protein function. It integrated protein 3D structures and protein language model (ESM-1b [31]) embeddings into a graph attention network [32] and optimized the model using the contrastive learning approach, which pulled the embeddings of the (anchor, positive) pair together and the embeddings of the (anchor, negative) pair away. By naturally representing the amino acids as nodes and spatial relations between residues as edges, the graph neural network can extract structural features in addition to sequence features and generate function-aware representations of the protein. In this work, we shifted from the multi-class single-label classification approach used in PenLight [14] to a multi-class multi-label classification framework, which better aligns with the function annotation data of enzymes in which an enzyme can be labeled with multiple EC numbers. PenLight2 achieved two key improvements compared to PenLight: model training (triplet sampling strategy) and model inference (function transfer scheme and prediction cutoff selection):

1) Triplet sampling strategy. For training efficiency, PenLight takes a multi-class single-label classification approach and randomly samples one EC number for promiscuous enzymes when constructing the triplet in contrastive learning, considering that only less than 10% enzymes in the database used are annotated with more than one EC number. To enhance the effectiveness of contrastive learning for promiscuous enzymes, in this work, we adopt a multi-class multi-label classification approach, in which retain the complete four-level EC number annotations for an enzyme in the triplet sampling of PenLight2 (Fig 1B). Specifically, we thus generalized PenLight’s hierarchical sampling scheme to accommodate proteins with multiple functions in PenLight2: in each training epoch, for each anchor protein (every protein in the training set), we randomly choose one of its ground truth EC numbers if it has more than one and then follow original sampling scheme in PenLight for the sampling of the positive and the negative proteins (S1 Text). A filter is applied to ensure that the anchor and the negative do not share EC numbers.

2) Function transfer scheme. The original PenLight used the pairwise embedding distance between the query protein and training proteins to identify the most similar protein (closest embedding distance) for transferring function annotations. We generalized PenLight’s protein-protein distance to a protein-cluster distance to improve the robustness in distance computation. Specifically, in PenLight2, we computed a cluster embedding ***z***_*γ*_ for an EC number *γ* by averaging all proteins with this EC number:

$$\begin{array}{c} z_{\gamma }=\frac{1}{\left|\{p:EC(p)=\gamma \}\right|}\sum_{p:EC(p)=\gamma }x_{p}, \end{array}$$ (5)

where *p* refers to a protein. Then, we computed the pairwise embedding distance between the query protein and all the EC clusters. We will transfer the EC numbers from annotated proteins to the query protein based on the computed distances. The smaller the distance with an EC cluster embedding is, the more likely that the query protein has this EC number. This approach, tailored for multi-label classification, creates a distance matrix between query proteins and EC numbers, which can be integrated seamlessly with our conformal prediction framework.

3) Prediction cutoff selection. In contrast to the original PenLight model that only predicted the top-1 EC number for a query protein, PenLight2 implemented an adaptive method to achieve multi-label EC prediction. Following the max-separation method proposed in our previous study [30], we sorted the distances between the query protein and all EC clusters and identified the max difference between adjacent distances. PenLight2 then uses the position with the max separation as the cutoff point and outputs all EC numbers before this point as final predictions. This cutoff selection method aligns with the multi-label nature of the task.

With these improvements, we extended the original PenLight from the single-label classification to the multi-label setting. We denote this improved version as PenLight2.

## Results

We performed multiple experiments to evaluate CPEC’s prediction accuracy and ability of FDR control. We further evaluated CPEC using test data that have low sequence identities to the training data to demonstrate its utility for generating hypotheses (function annotations) for novel protein sequences.

### CPEC achieves accurate enzyme function predictions

We first evaluated the prediction performance of PenLight2, the base ML model in CPEC, for predicting function annotations (EC numbers) of protein enzymes. The purpose of this experiment was to assess the baseline prediction accuracy of CPEC when the FDR control is not applied. We compared CPEC with three state-of-the-art deep learning methods capable of reliably predicting enzyme function on the fourth level of EC hierarchy, including two CNN-based (convolutional neural networks) methods DeepEC [9] and ProteInfer [11] that take protein sequence data as input and one GNN-based (graph neural networks) method DeepFRI [10] that takes both protein sequence and structure data as input. All these three methods applied the multi-class classification paradigm for function prediction: first predicting a score between 0 and 1 as the predicted probability that the input enzyme has a particular EC number and then generating all EC numbers with predicted probability greater than 0.5 (except for DeepFRI which used 0.1 as cutoff) as the final predicted function annotations for the input enzyme. We evaluated all methods using metrics F1 score, which assesses prediction accuracy considering both precision and recall, and the normalized discounted cumulative gain (nDCG) [33], which rewards higher rankings of true positives over false negatives in the prediction set (S1 Text). On a more challenging test set (test proteins with [0, 30%) sequence identity to training proteins), we further evaluated all methods by drawing the micro-averaged precision-recall curves.

**Fig 2:**
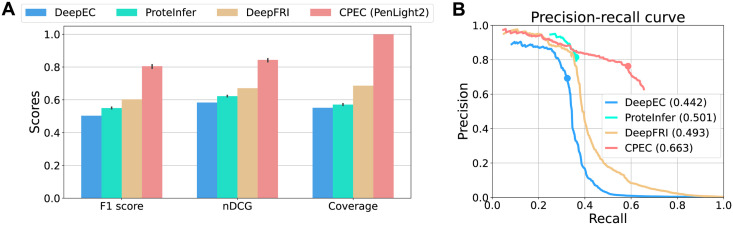
PenLight2, the base ML model of CPEC, outperforms the state-of-the-art methods for EC number prediction. (A) We evaluated DeepEC [9], ProteInfer [11], DeepFRI [10], and PenLight2 for predicting the 4th-level EC number, using F1 score, the normalized discounted cumulative gain (nDCG), and coverage as the metrics. Specifically, coverage is defined as the proportion of test proteins for which a method has made at least one EC number prediction. (B) We further evaluated all methods for predicting the 4th-level EC number on more challenging test proteins with [0, 30%) sequence identities to the training proteins and drew the micro-averaged precision-recall curves. For each curve, we labeled the point with the maximum F1 score (Fmax).

The evaluation results showed that our method outperformed all the three state-of-the-art methods in terms of both F1 score and nDCG (Fig 2A). For example, PenLight2 achieved a significant improvement of 34% and 26% for F1 and nDCG, respectively, over the second-best baseline DeepFRI. The pronounced performance gaps between PenLight2 and other baselines also suggested the effectiveness of the contrastive learning strategy used in PenLight2. The major reason is that contrastive learning utilized the structure of the function space (the hierarchical tree of the EC number classification system) to learn protein embeddings that reflect function similarity, while the multi-class classification strategy used in the three baselines just treated all EC numbers as a flat list of labels and may only capture sequence/structure similarity but not function similarity. In addition, we observed that methods that incorporated protein structure data (PenLight2 and DeepFRI) achieved better than methods that only use sequence data as input (DeepEC and ProteInfer), suggesting that protein structure may describe features related to functions more explicitly and is useful for predicting protein function. Those results demonstrated that the design choices of PenLight2, including the contrastive learning strategy and representation learning of protein structure, greatly improve the accuracy of protein function prediction. To further analyze PenLight2’s prediction performance, we delineated its F1 score into precision and recall and observed that PenLight2 has slightly lower precision than other methods but substantially higher recall and F1 score (S1 Fig). We noted that other baseline methods such as ProteInfer achieved the high precision score at a cost of low coverage (Fig 2A), meaning that they did not predict any functions for a large number of query proteins due to their high uncertainties in those proteins. Additionally, we evaluated PenLight despite that it only performs single-label prediction, and we found that PenLight and PenLight2 had similar performances. As the fraction of promiscuous enzymes is low in the test set, we expected PenLight2 to be a more accurate predictor than PenLight in future enzyme function prediction tasks when promiscuous enzymes prevail.

On a more challenging test set which only includes test proteins with [0, 30%) sequence identities to training proteins, we also observed that PenLight2 robustly predicted the EC numbers of the test proteins and outperformed all baseline methods (Fig 2B and S3 Fig). The improvement of the micro-averaged Fmax value from the best baseline method ProteInfer to PenLight2 was 32%. In the high recall region, PenLight2 achieved a higher precision value than any of the baseline methods. The results here were consistent with the results on the entire test set, which further proved the effectiveness of PenLight2 for EC number prediction.

### CPEC provides FDR control for EC number prediction

After validating its prediction performance, we integrated PenLight2 as the base model into the conformal prediction framework. Conformal prediction provides a flexible, data-driven way to find an optimal cutoff for PenLigth2 to decide whether to predict a function label for the input protein, such that the FDR on the test data is lower than the user-specified FDR upper bound *α*. Here, we performed experiments to investigate whether CPEC achieves the desired FDRs and how its prediction performance would change when varying *α*. For comparison, we compared CPEC to several other thresholding strategies for generating the prediction set, including 1) max-separation (Methods); 2) top-1, where only the EC number with the closest embedding distance to the input protein is predicted as output; and 3) *σ*-threshold, where all EC numbers with an embedding distance smaller than *μ* + 2*σ* to the input protein are predicted as output, where *μ* and *σ* are the mean and standard deviation of a positive control set that contains the distances between all true protein-EC number pairs. Platt scaling [34], a parametric calibration method, was further included as a thresholding strategy for comparison. We also included our baseline DeepFRI, which outputs EC numbers if it predicts that the probability of the input having this EC number is greater than a cutoff of 0.1. The purpose of the experiment here is not to show CPEC can outperform all other methods under all metrics but to show that CPEC can achieve a desired tradeoff by tuning the interpretable parameter *α* and simultaneously provide a rigorous statistical guarantee on its FDR. In an evaluation experiment, we have further compared CPEC with two point-uncertainty prediction methods (Monte Carlo dropout [35] and RED [36]), demonstrating that CPEC provides precise FDR control prior to validation, whereas MC dropout and RED can only evaluate FDR post-validation (S1 Text).

#### Reliable FDR controls

**Fig 3:**
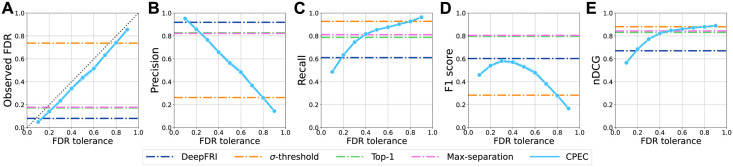
CPEC achieves FDR control for EC number prediction. For FDR tolerance *α* from 0.1 to 0.9 with increments 0.1, we evaluated how well CPEC controls the FDR for EC number prediction. Observed FDR risks, precision averaged over samples, recall averaged over samples, F1 score averaged over samples, and nDCG were reported for each FDR tolerance on test proteins in (A-E). The black dotted line in (A) represents the theoretical upper bound of FDR over test proteins. Three thresholding strategies were assessed over PenLight2 as a comparison to CPEC, which includes 1) max-separation [30], 2) top-1, and 3) *σ*-threshold. The results of CPEC were averaged over five different seeds. DeepFRI was also included for comparison.

In theory, the conformal prediction framework guarantees that the actual FDR of the base ML model on the test data is smaller than the pre-specified FDR level *α* with high probability. We first investigated how well this property holds on our function prediction task. We varied the value of *α* from 0 to 1, with increments of 0.1, and measured CPEC’s averaged per-protein FDR on the test data. As expected, we observed that the actual FDR of CPEC (Fig 3A, blue line) was strictly below the specified FDR upper bound *α* (Fig 3A, diagonal line) across different *α* values. This result suggested that CPEC successfully achieved reliable FDRs as guaranteed by the conformal prediction. We have further performed an evaluation experiment to investigate the impact of the calibration set sizes on CPEC’s FDR control, and the results suggested that the FDR control performances of CPEC were robust to various calibration set sizes (S1 Text).

#### Tradeoff between precision and recall with controlled FDR

Varying the FDR parameter *α* allowed us to trade-off between the prediction precision and recall of CPEC (Fig 3B and 3C). When *α* was small, CPEC predicted function labels for which it has the most confidence, in order to achieve a lower FDR, resulting in high precision scores (e.g., precision 0.9 when *α* = 0.1). When CPEC was allowed to tolerate a relatively larger FDR *α*, it predicted more potential function labels for the input protein at the FDR level it can afford, which resulted in an increasing recall score as *α* was increasing. Similarly, the nDCG score of CPEC was also increasing with *α* (Fig 3E), indicating that CPEC not only retrieved more true function labels but also ranked the true labels at the top of its prediction list.

#### Interpretable cutoff for guiding discovery

CPEC is able to compute an adaptive cutoff internally based on the user-specified FDR parameter *α* for deciding whether or not to assign a function label to the input protein. This allows researchers to prioritize or balance precision, recall, and FDR, depending on test budget or experiment expectations, in an ML-guided biological discovery process. In contrast, many existing methods that use a constant cutoff often have optimized performance in one metric but suffer in another. For example, in our experiment, DeepFRI and Platt scaling threshold strategy had the highest precisions but their recalls were the lowest among all methods; the *σ*-threshold strategy had a recall of 0.94 yet its FDR (0.75) was substantially higher than others (Fig 3A–3C and S2 Fig). Although some methods such as DeepFRI may achieve a better tradeoff between precision and recall by varying its probability cutoff from 0.1 to other values, they lack a rigorous statistical guarantee on the effect of varying the cutoff values. For example, if the cutoff of DeepFRI was raised from 0.1 to 0.9, one can expect that, qualitatively, it would lead to a higher precision but also a higher FDR. However, it is hard to quantitatively interpret the consequence of raising the cutoff to 0.9 (e.g., how high would the FDR be) until the model is evaluated using ground-truth labels, which are often unavailable before experimental validation in the process of biological discovery. In contrast, with CPEC, researchers are also able to balance the interplay between the prediction precision and recall by tuning the interpretable parameter *α* and assured that the resulting FDR will not be greater than *α*.

Overall, through these experiments, we validated that CPEC can achieve the statistical guarantee of FDR. We further evaluated the effect of varying the FDR tolerance *α* on CPEC’s prediction performances. Compared to conventional strategies for multi-label protein function prediction, CPEC provides a flexible and statistically rigorous way to better tradeoff precision and recall, which can be used to better guide exploration and exploitation in biological discovery with a controlled FDR.

### Adaptive prediction of EC numbers for proteins with different sequence identities to the training set

The risk in our conformal prediction framework is defined as the global average of per-protein FDRs, which may raise the concern that the overall FDR control achieved by CPEC on the test set was mainly contributed by FDR controls on those proteins that are easy to characterize and predict, and it is possible that the model suffered from pathological failures and did not give accurate FDR controls on proteins that are hard to predict. To this point, we defined the prediction difficulty based on the level of sequence identity between test proteins and training proteins, following the intuition that it is more challenging for an ML model to predict the functions of a protein if the protein does not have homologous sequences in the training data. We first performed a stratified evaluation to analyze CPEC’s FDR-control performance at different levels of prediction difficulty. After examining the consistency of the FDR control across different difficulties, we explored an adaptive strategy for predicting EC numbers, which allows the ML model not to predict a too specific EC number than what the evidence supported and only predict at the most confident level of EC hierarchy.

#### Consistency of FDR control

**Fig 4:**
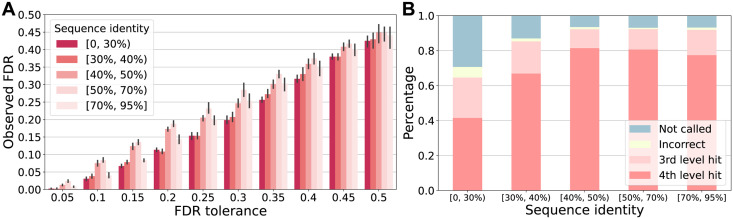
CPEC makes adaptive EC number predictions for proteins with different sequence identities to the training set. (A) We reported the observed FDR for test proteins with different sequence identities to the training set (i.e. different difficulty levels) for FDR tolerance *α* from 0.05 to 0.5 with increments of 0.05. Test proteins were divided into disjoint groups with [0, 30%), [30%, 40%), [40%, 50%), [50%, 70%), and [70%, 95%] sequence identity to the training set. The smaller the sequence identity, the harder the protein would be for machine learning models to predict function labels. (B) We designed the procedure to first predict the EC number at the 4th level. If the model was uncertain at this level and did not make any predictions, we would move to the 3rd level to make more confident conformal predictions instead of continuing with the 4th level with high risks. We used the same FDR tolerance of *α* = 0.2 for both levels of CPEC prediction. For proteins with different sequence identities to the training data, we reported the hit rate of our proposed procedure. The hit rate on the 4th level, the hit rate on the 3rd level, the percentage of proteins with incorrect predictions on both levels, and the percentage of not called proteins for both levels were reported. The results were calculated as an average over 5 different seeds of splitting the calibration set.

We first confirmed CPEC’s FDR-control ability across different levels of prediction difficulty. Specifically, we partitioned the test set into disjoint groups based on the following ranges of sequence identity to the training set: [0, 30%), [30%, 40%), [40%, 50%), [50%, 70%) and [70%, 95%]. We varied the values of *α* from 0.05 to 0.5 with increments of 0.05. For each level of FDR tolerance *α*, we examined the FDR within each group of test proteins. As shown in Fig 4A, CPEC achieved consistent FDR controls across different levels of train-test sequence identity and different values of *α*, where the observed FDR were all below the pre-specified FDR tolerance *α*. Even for the most difficult group of test proteins that only have [0, 30%) sequence identity to the training proteins, CPEC still achieved an FDR of 0.03 when tolerance *α* = 0.1. This is because a well-trained ML model would have low confidence when encountering difficult inputs, and CPEC would abstain from making predictions if the model’s confidence does not exceed the decision threshold. The results of this experiment built upon the conclusion of the previous subsection and validated that CPEC can not only control the FDR of the entire test set but also the FDR for each group of test proteins with different levels of prediction difficulty. We have performed an evaluation experiment to further assess CPEC’s FDR control on test proteins that do not belong to the same CATH superfamilies [37] as any of the training proteins. We found that CPEC provided effective FDR control for these test proteins from unseen superfamilies (S5–S7 Figs and S1 Text), suggesting that CPEC can offer effective FDR-controlled EC number predictions even for test proteins that are very dissimilar to its training proteins.

#### An adaptive strategy for EC number prediction

The EC number hierarchy assigns four-digit numbers to enzymes, where the 4th-level label describes the most specific functions of enzymes whereas the 1st-level label describes the most general functions. In EC number prediction, ideally, a predictive model should not predict a too specific EC number than what the evidence supported. In other words, if a model is only confident about its prediction up to the 3rd level of an EC number for a protein, it should not output an arbitrary prediction at the 4th level. We first trained two CPEC models, where the first model, denoted as CPEC4, predicts EC numbers at the 4th level as regular, and the other, denoted as CPEC3, predicts the 3rd-level EC numbers. We then combine the two models as a cascade model: given an input protein and a desired value of *α*, we first apply the CPEC4 to predict the 4th-level EC numbers for the input protein with an FDR at most *α*. If CPEC4 outputs any 4th-level EC numbers, they will be used as the fine-level annotations for the input; if CPEC4 predicts nothing due to the FDR tolerance *α* being too stringent, we apply CPEC3 on the same input to predict EC numbers at the 3rd-level. If CPEC3 outputs any 3rd-level EC numbers, they will be used as the coarse-level annotations for the input; otherwise, the cascade model just predicts nothing for this input. The motivation of this adaptive prediction strategy is that even though 3rd-level EC numbers are less informative than 4th-level ones, it might be more useful for researchers in certain circumstances to acquire confident 3rd-level EC numbers than only obtaining a prediction set with a large number of false positive EC numbers at the 4th level.

To validate the feasibility of the above adaptive model, we evaluated CPEC3 and CPEC4 using the same FDR tolerance *α* = 0.2 on our test set. We reported the hit rate, defined as the fraction proteins for which our model predicted at least one correct EC number, for both the 4th-level and the 3rd-level EC numbers. We found that this adaptive prediction model, compared to the model that only predicts at the 4th level, greatly reduced the number of proteins for which the model made incorrect predictions or did not make predictions (Fig 4B). For example, on the test group with sequence identity [0, 30%) to the training data, around 60% proteins were correctly annotated with at least one EC number, while only 40% proteins were correctly annotated if the adaptive strategy was not used. This experiment demonstrated the applicability of CPEC for balancing the prediction resolution and coverage in protein function annotation.

### Application: EC number annotation for low-sequence-identity proteins

Conformal prediction quantifies the ML model’s uncertainty in its predictions, especially for the predictions for previously unseen data. This is extremely useful in ML-guided biological discovery as we often need to make predictions for unseen data to gain novel discoveries. For example, in protein function annotation, the most challenging proteins to annotate are those previously uncharacterized or do not have sequence homologous in current databases. Conventional ML models that do not quantify prediction uncertainties are often overconfident when making predictions for the aforementioned challenging samples, leading to a large number of false positives in their predictions, which can incur a high cost in experimental validation without yielding a high true positive rate. Considering the importance of predicting previously uncharacterized data, here we designed an evaluation experiment to assess CPEC’s prediction performance on these challenging proteins. We created a test set that contains only proteins that have less than 30% sequence identity to any proteins in the training set, which simulated a challenging application scenario.

**Fig 5:**
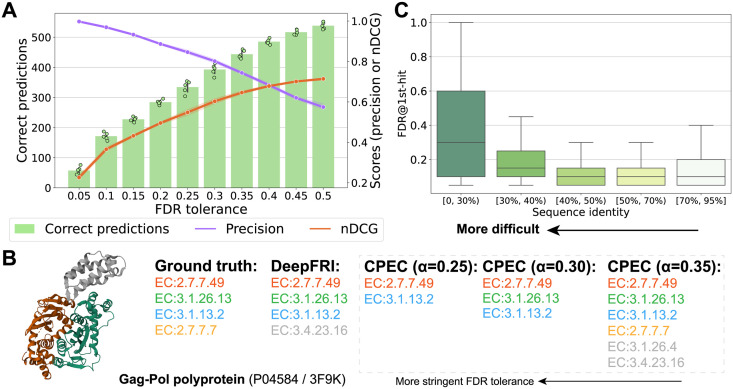
Application of FDR control for the EC number prediction of low-sequence-identity proteins. (A) CPEC was evaluated on difficult test proteins ([0, 30%) sequence identity to the training data). For FDR tolerance from 0.05 to 0.5, the total number of correct predictions, precision averaged over samples, and the normalized discounted cumulative gain was reported under five different seeds for splitting calibration data. Note that the upper bound of correct predictions, i.e. the ground truth labels, is 777. As a comparison, DeepFRI successfully made 307 predictions, with a sample-averaged precision of 0.8911 and an nDCG score of 0.5023. (B) An example of the prediction sets generated by CPEC for Gag-Pol polyprotein (UniProt ID: P04584; PDB ID: 3F9K), along with the prediction set from DeepFRI. CPEC used the chain A of the PDB structure as input. The prediction sets were generated under FDR tolerance *α* = 0.25, 0.3, 0.35. The sequence of this protein has [0, 30%) sequence identity to the training set and, therefore, can be viewed as a challenging sample. Incorrect EC number predictions are colored gray. (C) Boxplots showing the FDR@1st-hit metric, defined as the smallest FDR tolerance *α* at which CPEC made the first correct prediction for each protein. The evaluation was performed on five groups of test proteins, stratified based on their sequence identities to the training set.

We varied the FDR tolerance *α* from 0.05 to 0.5 and counted the number of correct predictions, where assigning one EC number to a protein was counted as one prediction. We observed that CPEC had an effective uncertainty quantification for its predictions on this low-sequence-identity test set (Fig 5A). For example, when *α* = 0.05 which forced the model to only output the most confident predictions, CPEC was highly accurate, with a precision of nearly 0.97. At the FDR tolerance level of 0.1, CPEC was able to retrieve 25% (180/777) true protein-EC number pairs at a precision higher than 0.9. Keeping increasing the value of *α* allowed CPEC to make more correct predictions, without significantly sacrificing precision. For instance, at the level *α* = 0.5, CPEC successfully predicted 70% true protein-EC number pairs while maintaining a reasonable precision of 0.6 and an nDCG of 0.7. As a comparison, the baseline method DeepFRI correctly predicted 309 protein-EC pairs, out of the total 777 true pairs, with a precision of 0.89 and an nDCG score of 0.50, which roughly corresponds to CPEC’s performance at *α* = 0.2.

We again note that CPEC is more flexible than methods such as DeepFRI in that it provides an interpretable and principled way to tradeoff between precision and recall, which allows researchers to not only prioritize high-confidence predictions but also increase prediction coverage for improving the yield rate of true positives. To illustrate this, we visualized the prediction results of CPEC and DeepFRI in Fig 5B. We selected a protein that has multiple EC number annotations (UniProt ID: P04584). Using its default setting, DeepFRI predicted four labels for this protein, among which three were correct. For CPEC, we gradually increased the value of *α* and see how the prediction set was changing. Interestingly, we observed that CPEC gradually predicted more true EC numbers as *α* was increasing while maintaining a low FDR. In particular, when *α* = 0.25, CPEC outputted two EC numbers, both of which were correct predictions; when *α* was relaxed to 0.3, CPEC predicted one more EC number, which turned out to be also correct; when we further relaxed the FDR tolerance *α* to 0.35, CPEC predicted six EC numbers for the protein, and four of them were correct. This example illustrated CPEC’s utility in practice: researchers have the flexibility when using CPEC to guide experiments, where a small value of *α* prioritizes accurate and confident hypotheses, and a large value of *α* promotes a high yield of true positives while ensuring the number of false positives to be limited.

Having observed that CPEC was able to recover more true function labels as we were relaxing the FDR tolerance *α*, we asked one important question—at which value of *α* can CPEC output the first correct function label (“hit”) for the input protein. We referred to this *α* value as FDR@1st-hit. This metric can be viewed as a proxy of the experiment cost researchers need to pay before they obtain the first validated hypothesis. We computed the FDR@1st-hit value for all test proteins in each of the five disjoint groups partitioned by their sequence identity to training sequences (Fig 5C). We found that for the majority of the test sequences (the four groups out of five with sequence identity at least 30% to training sequences), CPEC was able to reach the first hit at an FDR lower than 0.15. For the most difficult group where all proteins share [0, 30%) sequence identity to training data, the median FDR@1st-hit was 0.3. This observation was consistent with our intuition and expectation, as low-sequence-identity proteins are more difficult for the ML model to predict, thus requiring a larger hypotheses space to include at least one true positive. Overall, CPEC achieved a reasonable FDR@1st-hit for function annotation, meaning that it produced a limited number of false positives before recovering at least one true positive, which is a highly desired advantage in ML-guided biological discovery.

## Discussion

Machine learning models play a vital role in generating biological hypotheses for downstream experimental analyses and facilitating biological discoveries in various applications. A significant challenge in the process of ML-assisted biological discoveries is the development of ML models with interpretable uncertainty quantification of predictions. When applied to unseen situations, ML models without uncertainty quantification are susceptible to overconfident predictions, which misdirects experimental efforts and resources to the validation of false positive hypotheses. Addressing this challenge becomes essential to ensure the efficiency and reliability of ML-assisted biological discovery.

In this work, we have presented CPEC, an ML framework that enables FDR-controlled ML-assisted biological discoveries. Leveraging the conformal prediction framework, CPEC allows users to specify their desired FDR tolerance *α*, tailored to the experiment budget or goals and makes corresponding predictions with a controlled FDR. We demonstrate CPEC’s effectiveness using enzyme function annotation as a case study, simulating the discovery process of identifying the functions of less-characterized enzymes. PenLight2, an improved version of PenLight optimized for multi-label classification is utilized as CPEC’s base ML model. Specifically, CPEC takes the sequence and structure of an enzyme as input and outputs a set of functions (EC numbers) that the enzyme potentially has. The conformal prediction algorithm in CPEC theoretically guarantees that the FDR of the predicted set of functions will not exceed *α* with high probability. The evaluation of CPEC on the EC number prediction task showed that CPEC provides reliable FDR control and has comparable or better prediction accuracy than baseline methods at a much lower FDR. Interpretable cutoffs were provided by CPEC for guiding the EC number annotations of proteins. Furthermore, CPEC demonstrated its robustness in making FDR-controlled predictions even for proteins with low sequence identity to its training set.

Quantifying uncertainties of ML model predictions is a key desideratum in ML-guided biological discovery. Although a few prior studies have investigated the uncertainty quantification of ML models [12, 38], their uncertainty estimates are only indicative of prediction errors but do not translate to error-controlled predictions. In contrast, CPEC enables researchers to specify a maximum level of error rate and produces a set of predictions whose error rate is guaranteed to be lower than the specified level. Additionally, CPEC stands out by providing risk estimates, which delivers insights into the potential outcomes even before experimental validation and aids in the strategic allocation of experimental resources. One limitation of the CPEC framework is that when under covariate shift (i.e., *P*_*calib*_(*X*) ≠ *P*_*test*_(*X*)), the data assumption of CPEC that the data in the calibration and test sets are *i.i.d.* is violated, which might lead to suboptimal FDR control performances (S6 and S7 Figs). Although weighted conformal prediction frameworks have been proposed to address this limitation [39], the quantification and control of non-monotonic risk functions (e.g., FDR) under covariate shift remained a challenging problem. In this work, we define the error rate as the false discovery rate (FDR) to reflect the practical consideration in experiments where the goal is to maximize the success rate of hypothesis validation given a limited test budget. Nevertheless, the CPEC framework can be extended to support other forms of error rates, such as false negative rate [13]. In addition to protein function annotation, we expect CPEC to be a valuable tool for researchers in other biological discovery applications particularly when a balance between the experimental budget and the high yield rate is desired, such as drug target identification [40], material discovery [41], and virtual molecule screening [38].

## Supporting information

S1 Text Supplementary information. Additional methodology, detailed experiment descriptions, and further evaluation experiments are included in the file.
(PDF)

S1 Fig Performance evaluation of representative baseline methods for EC number prediction. We evaluated DeepEC, ProteInfer, DeepFRI, and CPEC (PenLight2) for predicting the 4th level EC number, using sample-averaged precision and recall as the metrics. DeepEC and DeepFRI were evaluated using the only trained model provided in their repositories, whereas ProteInfer was assessed using 5 different trained models. DeepFRI was trained on the same dataset as PenLight2 while DeepEC and ProteInfer were trained by their respective datasets. PenLight2 was trained using 5 different seeds.
(PDF)

S2 Fig CPEC achieves FDR control for EC number prediction. Platt scaling [34], RED [36], and Monte Carlo dropout [35] were further evaluated as thresholding strategies, in comparison to CPEC. Due to the requirements of the methods, RED and MC dropout were applied on top of an MLP model. The results of CPEC and all of the thresholding strategies were averaged over five different seeds.
(PDF)

S3 Fig Performance evaluation of representative baseline methods for EC number prediction on test proteins with [0, 30%) sequence identities to training proteins. We evaluated DeepEC, ProteInfer, DeepFRI, and CPEC (PenLight2) for predicting the 4th level EC number, using sample-averaged precision, recall, F1 score, nDCG, and coverage as the metrics. DeepEC and DeepFRI were evaluated using the only trained model provided in their repositories, whereas ProteInfer was assessed using 5 different trained models. DeepFRI was trained on the same dataset as PenLight2 while DeepEC and ProteInfer were trained by their respective datasets. PenLight2 was trained using 5 different seeds.
(PDF)

S4 Fig The FDR control of CPEC with different calibration set sizes. The performances of CPEC’s FDR control were evaluated using calibration sets with various sizes (abbrev: calib. set size): 20%, 10%, 5%, and 1% of the total number of the training data. The same training data was used across all calibration set sizes to ensure consistency in the comparison. The black dotted line in the first panel represents the theoretical upper bound of FDR over test proteins. The results were averaged over five different seeds.
(PDF)

S5 Fig Performance evaluation of representative baseline methods for EC number prediction on test proteins that do not belong to the same CATH [37] superfamilies as the training proteins. CPEC and three baseline methods (DeepEC, ProteInfer, and DeepFRI) were evaluated for predicting the 4th level EC number, using sample-averaged precision, recall, F1 score, nDCG, and coverage as the metrics. DeepEC and DeepFRI were evaluated using the only trained model provided in their repositories, whereas ProteInfer was assessed using 5 different trained models. DeepFRI was trained on the same dataset as CPEC, while DeepEC and ProteInfer were trained using their respective datasets. Training proteins not labeled in the CATH database were only removed from the training dataset of CPEC but not from the baseline methods’ training sets, which gave potential advantages to baseline methods. CPEC was trained using 5 different seeds.
(PDF)

S6 Fig Application of the FDR control for the EC number prediction of out-of-distribution (OOD) proteins. The FDR control of CPEC was evaluated on a more challenging data split: no training and test proteins belong to the same CATH [37] superfamily. Training proteins not labeled in the CATH database were only removed from the training dataset of CPEC but not from the baseline methods’ training sets, which gave potential advantages to baseline methods. The results were averaged over five different seeds.
(PDF)

S7 Fig Application of the FDR control for the EC number prediction of out-of-distribution (OOD) proteins. The FDR control of CPEC was evaluated on a more challenging data split: no training and test proteins belong to the same CATH [37] superfamily. A total number of 200 test proteins were sampled from the test set, and proteins that belong to the same superfamilies as the sampled test proteins were removed from the training set of CPEC. Training proteins not labeled in the CATH database were only removed from the training dataset of CPEC but not from the baseline methods’ training sets, which gave potential advantages to baseline methods. The results were averaged over five different seeds.
(PDF)

S8 Fig PenLight2, the base ML model of CPEC, outperforms the state-of-the-art methods for EC number prediction. CPEC (PenLight2) and four baseline methods (including a baseline MLP model that takes the ESM-1b protein embeddings as the input) were evaluated for predicting the 4th-level EC number on more challenging test proteins with [0, 30%) sequence identities to the training proteins and the micro-averaged precision-recall curves were drawn. For each curve, the point with the maximum F1 score (Fmax) was labeled.
(PDF)

S9 Fig Evaluation of two point-uncertainty prediction approaches. Two point-uncertainty prediction methods (Monte Carlo dropout (MC dropout) [35] and RED [36]) were evaluated in terms of uncertainty quantification. To make a fair comparison, a multi-layer perception taking ESM-1b protein embedding as the input was selected as the base ML model. The percentiles of the prediction variance (10th, 20th, 30th,…, and 100th percentiles) on the test set were used as the cutoffs. Predictions with variances larger than the cutoff were dropped. Observed false discovery rate (FDR), precision, recall, and coverage were used as metrics. The results were averaged over five different seeds.
(PDF)
