# Supplementary material for: Leveraging conformal prediction to annotate enzyme function space with limited false positives
Source: PLoS Comput Biol. 2024 May 29;20(5):e1012135. doi: 10.1371/journal.pcbi.1012135 (PMC11164347; doi:10.1371/journal.pcbi.1012135)
Supplement: S1 Text — Additional methodology, detailed experiment descriptions, and further evaluation experiments are included in the file. (PDF) [file pcbi.1012135.s001.pdf]

Supplementary Information

Conformal risk control theory

Conformal risk control guarantees

For conformal risk control, different forms of guarantees can be selected based on the specific characteristics of each task. One commonly used type of guarantees, *conformal risk control guarantee* [1], is shown as follows:

$$\mathbb{E}[l(C_\lambda(X^t), Y^t)] \le \alpha, \tag{S1}$$

where l refers to a monotonically non-increasing loss function with respect to λ, $C_\lambda(\cdot)$ refers to the function of generating the prediction set induced by λ, and α is the user-defined risk threshold. In applications, monotonic loss functions such as miscoverage and false negative rate (FNR) are used [2]. Specifically, the guarantee using miscoverage as the loss function, also known as the *conformal coverage guarantee* [3], takes the below form:

$$\mathbb{E}[\mathbb{1}\{Y^t \notin C_\lambda(X^t)\}] = \mathbb{P}(Y^t \notin C_\lambda(X^t)) \le \alpha. \tag{S2}$$

Calibration algorithm proofs

In the calibration step of CPEC, we adopt the LTT algorithm for achieving FDR control. For each candidate model parameter λ, we calculated a p-value for the null hypothesis $\mathcal{H} : R(\lambda) > \alpha$. We used Hoeffding's inequality p-values for hypothesis testing, and the proofs of the validity of the Hoeffding's inequality p-values is provided as below.

For n independent random variables $X_1, \ldots, X_n$, each of which is bounded in the form of $a_i \le X_i \le b_i$, Hoeffding shows that the following inequality holds [4]:

$$\mathbb{P}(|\bar{X} - \mu| \ge t) \le 2\exp\left\{\frac{-2(nt)^2}{\sum_{i=1}^{n}(b_i - a_i)^2}\right\}, \tag{S3}$$

where $\bar{X} = \frac{1}{n}\sum_{i=1}^{n} X_i$ and $\mu = \mathbb{E}\left[\bar{X}\right]$. This inequality is known as the Hoeffding's inequality. Hoeffding also proves that the one-sided inequality holds. When the upper bounds and the lower bounds of X_i are same across different variables, the one-sided inequality can be formulated as below:

$$\begin{cases} \mathbb{P}(\bar{X} - \mu \ge t) \le \exp\left\{\frac{-2nt^2}{(b-a)^2}\right\} \\ \mathbb{P}(\bar{X} - \mu \le -t) \le \exp\left\{\frac{-2nt^2}{(b-a)^2}\right\} \end{cases}, \tag{S4}$$

where $0 < t < b - \mu$.

The above Hoeffding's inequality allows us to develop a valid p-value for testing the null hypothesis regarding FDR control. As the FDR value is bounded by 0 and 1, the derivation of the p-value is as below [5]:

$$\begin{aligned} \mathbb{P}(R(\lambda) > \alpha) =& \mathbb{P}(\hat{R}(\lambda) - R(\lambda) < -(\alpha - \hat{R}(\lambda))) \\ &\le \exp(-2n(\alpha - \hat{R}(\lambda))_+^2) = p_\lambda^{\text{Hoeffding}}. \end{aligned} \tag{S5}$$

When $p_\lambda^{\text{Hoeffding}} \le \delta$, it means that the FDR control guarantee is satisfied and this λ is a valid model parameter. Hence, we use $p_\lambda^{\text{Hoeffding}}$ as the p-value for hypothesis testing in CPEC.

Baseline implementation

For performance evaluation, we used four baselines for EC number prediction: DeepEC [6], ProteInfer [7], DeepFRI [8] and PenLight [9]. As EC numbers are the most specific on the fourth-level, we only focused on EC number prediction methods that can reliably predict on the fourth-level of EC hierarchy. The implementation details of the baselines are in the following:

DeepEC: DeepEC is a CNN-based protein sequence model trained by supervised learning. The backbone of the model consisted of three convolutional neural networks (CNNs) which made predictions about whether the input protein is an enzyme, the third-level EC digits, and the fourth-level EC digits, respectively. DeepEC was trained on $1,388,606$ protein sequences from the datasets Swiss-Prot [10] and TrEMBL [11]. As the script for training is not provided, we used the only trained model in the repository of DeepEC and ran it on our test set to make EC number predictions.

ProteInfer: ProteInfer is also a CNN-based model for protein function predictions. It used one-hot encoding to represent protein sequences followed by a series of residual dilated convolutional blocks. The per-residue embeddings outputted by the convolutional layers then went through a mean-pooling layer and mapped to probabilities of possible class labels. ProteInfer was trained on Swiss-Prot dataset ([10]) via supervised training. It used two different data splitting methods: The first is to randomly split the dataset into train, test, and validation sets at approximately $8:1:1$ ratio, resulting in $438,522$ sequences for training; The second is to use UniRef50 clustering to do the data splitting by setting the maximum sequence similarity between the training and test sets, which yielded $182,965$ training sequences. Due to the restriction of computational resources to train ProteInfer, we evaluated the performance of ProteInfer on our test set using 5 different trained models under random split from their repository.

DeepFRI: DeepFRI is a graph convolutional network (GCN) that integrated protein sequence features from a language model and the backbone structure features. DeepFRI utilized a Long-Short-Term-Memory (LSTM) language model to process protein sequences and extract sequence embeddings, which served as node embeddings in the GCN. The GCN consisted of three graph convolutional layers, which took the contact map of all the C_α atoms in the protein as input and were optimized to learn the structure information via supervised learning. We used the GPU version trained model in DeepFRI's repository and used the PDB files of our test set as input to conduct EC number prediction.

PenLight: PenLight [9] is a structure-based graph attention network model for protein function predictions. PenLight model was trained using contrastive learning which utilized hierarchically sampled protein triplets as training data. PenLight leveraged both the sequence information from protein language models (pLMs: e.g., ESM-1b [12]) and the structure information captured by the graph attention network. Specifically, PenLight used a hierarchical sampling scheme to sample the triplets of (anchor, positive, negative) from the original dataset: the positive sample shares the same EC number as the anchor while the negative sample does not. Through training, the learned embeddings of the (anchor, positive) pair would be pulled together and the embeddings of the (anchor, negative) pair would be pulled away. Each protein in the training set with EC number $a.b.c.d$ is set as the anchor, and a similarity level $\beta \in \{1,2,3,4\}$ is randomly selected. Another protein in the training set with the first β EC digits the same as the anchor would be sampled as the positive, and a different protein in the training set with only the first $\beta-1$ EC digits the same as the anchor would be sampled as the negative. To avoid the negative impact on training

by too trivial or too hard protein pairs, PenLight utilized a technique called hard positive/negative mining on top of the hierarchical sampling. During training in each mini-batch, PenLight applied hierarchical sampling to all proteins in the triplets with an additional criterion that the positive protein has the largest distance from the anchor in all the possible positives and the negative has the smallest. Finally, PenLight adopted the soft margin loss as the training objective, which takes the below form:

$$\mathcal{L}(\mathbf{x}_{\mathrm{anc}},\mathbf{x}_{\mathrm{pos}},\mathbf{x}_{\mathrm{neg}}) = \frac{1}{n}\sum_{i=1}^{n}\log\left(1+\exp(d(\mathbf{x}_{\mathrm{anc}}^{(i)},\mathbf{x}_{\mathrm{pos}}^{(i)}) - d(\mathbf{x}_{\mathrm{anc}}^{(i)},\mathbf{x}_{\mathrm{neg}}^{(i)})\right), \quad \textbf{(S6)}$$

where $\mathbf{x}_{\mathrm{anc}},\mathbf{x}_{\mathrm{pos}},\mathbf{x}_{\mathrm{neg}}$ represent the embeddings of the anchor protein, the positive protein, and the negative protein respectively, $d(\cdot,\cdot)$ refers to the Euclidean distance, and n refers to the dimension of the output embeddings.

PenLight2: For performance evaluation, we trained PenLight2 under 5 different seeds, made predictions using the max separation method [13], and evaluated its performance on the test set.

Evaluation

Normalized Discounted Cumulative Gain: We adopted the Normalized Discounted Cumulative Gain (nDCG) as one of our evaluation metrics. To compute the nDCG value of our prediction results (a list of probabilities representing how likely the query protein has the corresponding EC number), first we need to calculate the Discounted Cumulative Gain (DCG) and the Ideal Discounted Cumulative Gain (IDCG) at a particular rank position k. Suppose the number of all possible predicted EC numbers is n, the ground truth probabilities sorted in descending order are $\{q_i\}$ ($q_i = 0$ or 1 depending on whether the query protein has the ith EC number), and the ground truth probabilities sorted in the order induced by the predicted probabilities (also in descending order) are $\{p_i\}$, the DCG and IDCG are computed as follows:

$$DCG = \sum_{i=1}^{n}\frac{p_i}{\log_2(i+1)},\ IDCG = \sum_{i=1}^{n}\frac{q_i}{\log_2(i+1)}, \quad \textbf{(S7)}$$

where n is the number of all possible EC labels predicted by the model, p_i is the predicted probability for the ith EC number outputted by the model, and $q_i = 1$ for $i \leq t$ and $q_i = 0$ otherwise (t is the number of ground truth EC labels that the query protein has). For each protein, EC labels with the same predicted probabilities are considered as ties. nDCG is computed as follows:

$$nDCG = \frac{DCG}{IDCG}. \quad \textbf{(S8)}$$

Since nDCG is a normalized value between 0 and 1, it could serve as a good evaluation metric across different models.

Comparisons between conformal prediction and other baseline strategies

As PenLight2 is a contrastive learning-based method, it originally returns a distance value for each pair of protein and EC number, where a smaller distance implies a higher probability of the protein having that EC number. We negated the distance value and linearly rescaled them into $[0,1]$ through min-max normalization, where we set min as the opposite number of the max distance on training data and max as 0, to conform with the general ML classification methods. For experiments involving CPEC, we all performed the above-mentioned transform process for PenLight2 predictions. The calibration set was randomly constructed out of the original training data and was excluded during training. The calibration set is of 10% the size as the original training set. Notably, when splitting the

calibration set from the original training set, we performed iterative train test splits [14, 15], a method for multi-label data stratification, which ensures that all EC labels are preserved in the new training set. For each FDR tolerance α, we carried out CPEC to acquire the valid model parameter λ and generated the corresponding prediction sets. We averaged our results over five different seeds for splitting the calibration set for all experiments.

Platt scaling. Apart from the three thresholding strategies shown in Fig 3, we further compared CPEC to Platt scaling [16], which is a parametric calibration method to transform the outputs of classification models into probability distribution. In our case, we leveraged Platt scaling to transform the distance calculated by PenLight2 into the probability distribution over EC numbers. The transformation is formulated as below:

$$\mathbb{P}(y=1|x) = \frac{1}{1+\exp(Ad(x,y)+B)}, \tag{S9}$$

where A, B are scalar parameters, and $d(x,y)$ is the Euclidean distance between protein and EC number embeddings. Specifically, we formulated the multi-class multi-label classification problem as a binary classification problem for each pair of protein and EC number. We trained the logistic regression calibration model of Platt scaling on the calibration set using NLL loss [17] and performed classification on the test set, identical to that of CPEC. The classifcation on calibrated test set predictions used 0.5 as the cutoff. The results are shown in Fig S2.

Monte Carlo dropout and RED. Both Monte Carlo dropout [18] and RED [19] are point-uncertainty prediction methods. Given a trained ML model for classification tasks and a test input, both approaches will provide the mean and variance of the predicted probabilities for the input. We conducted an evaluation experiment to evaluate and compare the calibration and uncertainty quantification capabilities of CPEC with those of RED and MC Dropout. As RED operates on class probability outputs (i.e., normalized to have a sum of 1 across all classes) from the SoftMax layer in a neural network, it is not straightforward to translate the distance predictions of CPEC (PenLight2) into normalized class probabilities. Similarly, MC dropout is also tailored to function with direct probability outputs from neural network models. Therefore, we have trained a variant of CPEC – an MLP-based ML model that takes the ESM-1b protein embeddings as input, which allows us to apply RED and MC dropout effectively for uncertainty quantification. The MLP model consists of two hidden layers (with dimensions of 1000 and 512, respectively) and a prediction layer that maps the hidden embeddings to logits for each EC number. We used ReLU as the activation function for each hidden layer and used a dropout rate of 0.5. During training, we used the Adam optimizer and cross-entropy loss function, setting the learning rate as 0.001, the weight decay as 0.0001, and the batch size as 512. In addition, we used the validation loss for early stopping with a patience of 20 epochs.

We first validated the efficacy of our trained MLP model by evaluating its predictive performance on a test set with proteins with less than 30% sequence identities to the training proteins. The performance of our MLP model was found to be on par with our baseline methods (i.e., DeepEC, ProteInfer, and DeepFRI) (Fig S8), thus validating its suitability for subsequent application of RED and MC Dropout. Next, we evaluated the calibration capabilities of RED and MC dropout by evaluating their calibrated predicted probabilities (the mean of the predicted probabilities). The results showed that, despite a slight improvement in the F1 score with MC dropout (Fig S2), both RED and MC dropout demonstrated similar EC number prediction performances to the base MLP model, indicating that the calibration of RED and MC dropout only slightly improves the predictive performance of our MLP model.

Furthermore, we have designed an experiment to leverage the predicted uncertainties of MC dropout and RED to guide the predictions of EC numbers. As both approaches output the variance of the predicted probabilities, we

used the quantiles of the variances on all test proteins as the cutoff: the model will only make predictions when the variance is lower than the cutoff. Intuitively, a lower quantile cutoff corresponds to a more conservative strategy (only predicting when highly confident), and an upper quantile cutoff corresponds to a less restrictive strategy (making predictions even when there is uncertainty). We have experimented with 10 percentiles (10th, 20th, ..., 100th). We observed that varying the quantile cutoffs provided tradeoffs between high precision (low FDR) and low precision (high FDR). However, unlike CPEC, which provides precisely controlled precision and FDR using α (Fig 3), the relationship between the quantile and the precision/FDR for both MC dropout and RED is not linear (Fig S9), and therefore it is unclear how to select a suitable quantile cutoff for a given FDR tolerance before the validation. This observation suggested that CPEC provides precise FDR control prior to validation, whereas MC dropout and RED can only evaluate FDR post-validation.

Impact of calibration set sizes on the FDR control of CPEC

To investigate the impact of the calibration set size on the False Discovery Rate (FDR) control in our CPEC method, we have conducted an evaluation experiment using various calibration set sizes, set to 20%, 10%, 5%, and 1% of the total training data, respectively. We varied FDR tolerance α from 0 to 1, with increments of 0.1, and evaluated CPEC's per-protein FDR with different calibration set sizes on the test data. To maintain a consistent basis for comparison, the same training dataset was utilized across all different calibration set sizes. This approach reflects the real-world scenario where, given a pre-trained machine learning model, one aims to determine the most appropriate calibration set size.

We observed that CPEC provided robust and reliable FDR control across various calibration set sizes (Fig S4). For all calibration set sizes examined, the observed per-protein FDR consistently remained below the theoretical upper bound of FDR on the test proteins, indicating an effective FDR control of the model's predictions. We found that the smallest calibration size (1% of the training data) is the most challenging case, where the curve of observed FDR v.s. FDR tolerance deviated from the diagonal (perfect calibration). This was expected because the small calibration set did not include a sufficient number of proteins that well represent the true data distributions, thereby leading to inaccurate calibration. However, as the calibration set size increased, the observed FDR of CPEC on the test proteins approached the theoretical upper bound (diagonal line in Fig S4). We further observed that there was no significant changes in the observed FDRs when we varied the calibration set size in the range of 5% to 20% of the total training data (approximately 500 to 2000 calibration proteins), suggesting that the performance of CPEC's FDR control was robust once the calibration set reached to a reasonable size (e.g., $>$5% of the training data size).

We note that reserving $\sim$10% of training data for calibration purpose does not limit the applicability of CPEC, as common practices in machine learning research for computational biology also recommend withholding $\sim$10% of training data as validation set to tune hyperparameters [20].

Evaluation of CPEC on CATH-superfamily-based test split

We have performed an evaluation experiment to examine CPEC's performances in a more challenging setting, where all test proteins belong to different CATH superfamilies [21] from those of the training proteins. From the results, we observed that CPEC still provided reliable FDR control on this more challenging test setting, although less effective as compared to low-sequence-identity test set (test proteins with no more than 30% sequence identities to the

training proteins).

Data preprocessing and dataset splits. The CATH database is an expert-curated database that classifies 3D protein structures from the Protein Data Bank [22] into a hierarchical classification system: Class (C), Architecture (A), Topology (T), and Homologous superfamily (H). For the proteins in our dataset, we extracted their CATH codes directly from the CATH database. Out of the total 10,245 proteins in our dataset, 8,304 proteins were assigned CATH codes. We have created two datasets based on CATH superfamilies to evaluate the FDR control of CPEC. In the first dataset, we kept the training set from the original low-sequence-identity split but only filtered the test set such that no test protein shared the same CATH superfamily with any training protein. This resulted in a restricted test set with 48 proteins (test set A). To prevent data leakage, proteins that were not labeled in the CATH database were removed from the training set of CPEC. For baseline methods, as we used the trained models released by the authors, their training data still included those training proteins without CATH labels, which gave potential advantages to baseline methods. In the second dataset, we created a larger test set of 200 test proteins (test set B) by randomly sampling 200 proteins from the original test set from the low-sequence-identity split and further removed training proteins that belonged to the same superfamily as any of the 200 sampled test proteins. This led to a reduced training set of $\sim$2,500 proteins (the original training set contains $\sim$8,000 proteins). We next compared CPEC and baseline methods on these two datasets.

CPEC achieves accurate EC number prediction and provides reliable FDR control. First, we observed that CPEC outperformed baseline methods in predicting EC numbers on test set A (Fig S5). Subsequently, we found that CPEC also effectively achieved FDR control on test set A (Fig S6), where the observed FDR on the test proteins consistently remained below the theoretical upper bound of FDR. It is noteworthy, however, that CPEC's FDR control on this subset was less accurate than on the entire test set (Figs S6 and S4), where the curves of observed FDR and FDR tolerance did not exhibit a linear relationship as in Fig S4. This was particularly evident for FDR tolerance α ranging from 0.2 to 0.7, where the observed FDR significantly diverged from the theoretical upper bound. This pattern of results was also observed on test set B (Fig S7). The primary reason was that the test proteins in this evaluation, following the CATH-superfamily split, were more dissimilar to the training proteins as compared to the previously sequence-identity-based split, which was more challenging for CPEC to predict and calibrate. Nevertheless, the evaluation results the observed FDRs of CPEC still remain below the user-specified FDR tolerance levels (Fig S7), suggesting that CPEC can offer effective FDR-controlled EC number predictions even for test proteins that are very dissimilar to its training proteins.

We conclude that CPEC effectively maintained the FDR on these more challenging test sets below the user-specified FDR tolerance α. This conclusion suggested CPEC's robustness and reliability in adhering to FDR constraints, even in more challenging testing scenarios.

References

1. Angelopoulos, A. N., Bates, S., Fisch, A., Lei, L. & Schuster, T. Conformal risk control. *arXiv preprint arXiv:2208.02814* (2022).
2. Angelopoulos, A. N. & Bates, S. A gentle introduction to conformal prediction and distribution-free uncertainty quantification. *arXiv preprint arXiv:2107.07511* (2021).
3. Vovk, V., Gammerman, A. & Saunders, C. Machine-learning applications of algorithmic randomness (1999).
4. Hoeffding, W. in *The collected works of Wassily Hoeffding* 409–426 (Springer, 1994).
5. Bates, S., Angelopoulos, A., Lei, L., Malik, J. & Jordan, M. Distribution-free, risk-controlling prediction sets. *Journal of the ACM (JACM)* **68,** 1–34 (2021).
6. Ryu, J. Y., Kim, H. U. & Lee, S. Y. Deep learning enables high-quality and high-throughput prediction of enzyme commission numbers. *Proceedings of the National Academy of Sciences* **116,** 13996–14001 (2019).
7. Sanderson, T., Bileschi, M. L., Belanger, D. & Colwell, L. J. ProteInfer: deep networks for protein functional inference. *Biorxiv* (2021).
8. Gligorijević, V. *et al.* Structure-based protein function prediction using graph convolutional networks. *Nature communications* **12,** 1–14 (2021).
9. Luo, J. & Luo, Y. *Contrastive learning of protein representations with graph neural networks for structural and functional annotations* in *PACIFIC SYMPOSIUM ON BIOCOMPUTING 2023: Kohala Coast, Hawaii, USA, 3–7 January 2023* (2022), 109–120.
10. Bairoch, A. & Apweiler, R. The SWISS-PROT protein sequence database and its supplement TrEMBL in 2000. *Nucleic acids research* **28,** 45–48 (2000).
11. Consortium, U. UniProt: a hub for protein information. *Nucleic acids research* **43,** D204–D212 (2015).
12. Rives, A. *et al.* Biological Structure and Function Emerge from Scaling Unsupervised Learning to 250 Million Protein Sequences. *PNAS.* https://www.biorxiv.org/content/10.1101/622803v4 (2019).
13. Yu, T. *et al.* Enzyme function prediction using contrastive learning. *Science* **379,** 1358–1363 (2023).
14. Sechidis, K., Tsoumakas, G. & Vlahavas, I. *On the stratification of multi-label data* in *Joint European Conference on Machine Learning and Knowledge Discovery in Databases* (2011), 145–158.
15. Szymański, P. & Kajdanowicz, T. *A network perspective on stratification of multi-label data* in *First International Workshop on Learning with Imbalanced Domains: Theory and Applications* (2017), 22–35.
16. Platt, J. *et al.* Probabilistic outputs for support vector machines and comparisons to regularized likelihood methods. *Advances in large margin classifiers* **10,** 61–74 (1999).
17. Guo, C. *et al. On calibration of modern neural networks* in *International conference on machine learning* (2017), 1321–1330.
18. Gal, Y. & Ghahramani, Z. *Dropout as a bayesian approximation: Representing model uncertainty in deep learning* in *international conference on machine learning* (2016), 1050–1059.
19. Qiu, X. & Miikkulainen, R. *Detecting misclassification errors in neural networks with a gaussian process model* in *Proceedings of the AAAI Conference on Artificial Intelligence* **36** (2022), 8017–8027.
20. Lee, B. D. *et al.* Ten quick tips for deep learning in biology. *PLoS computational biology* **18,** e1009803 (2022).
21. Sillitoe, I. *et al.* CATH: increased structural coverage of functional space. *Nucleic acids research* **49,** D266–D273 (2021).
22. Berman, H. M. *et al.* The protein data bank. *Nucleic acids research* **28,** 235–242 (2000).
