# Supplementary figures and images for: Leveraging conformal prediction to annotate enzyme function space with limited false positives
Source: PLoS Comput Biol. 2024 May 29;20(5):e1012135. doi: 10.1371/journal.pcbi.1012135 (PMC11164347; doi:10.1371/journal.pcbi.1012135)

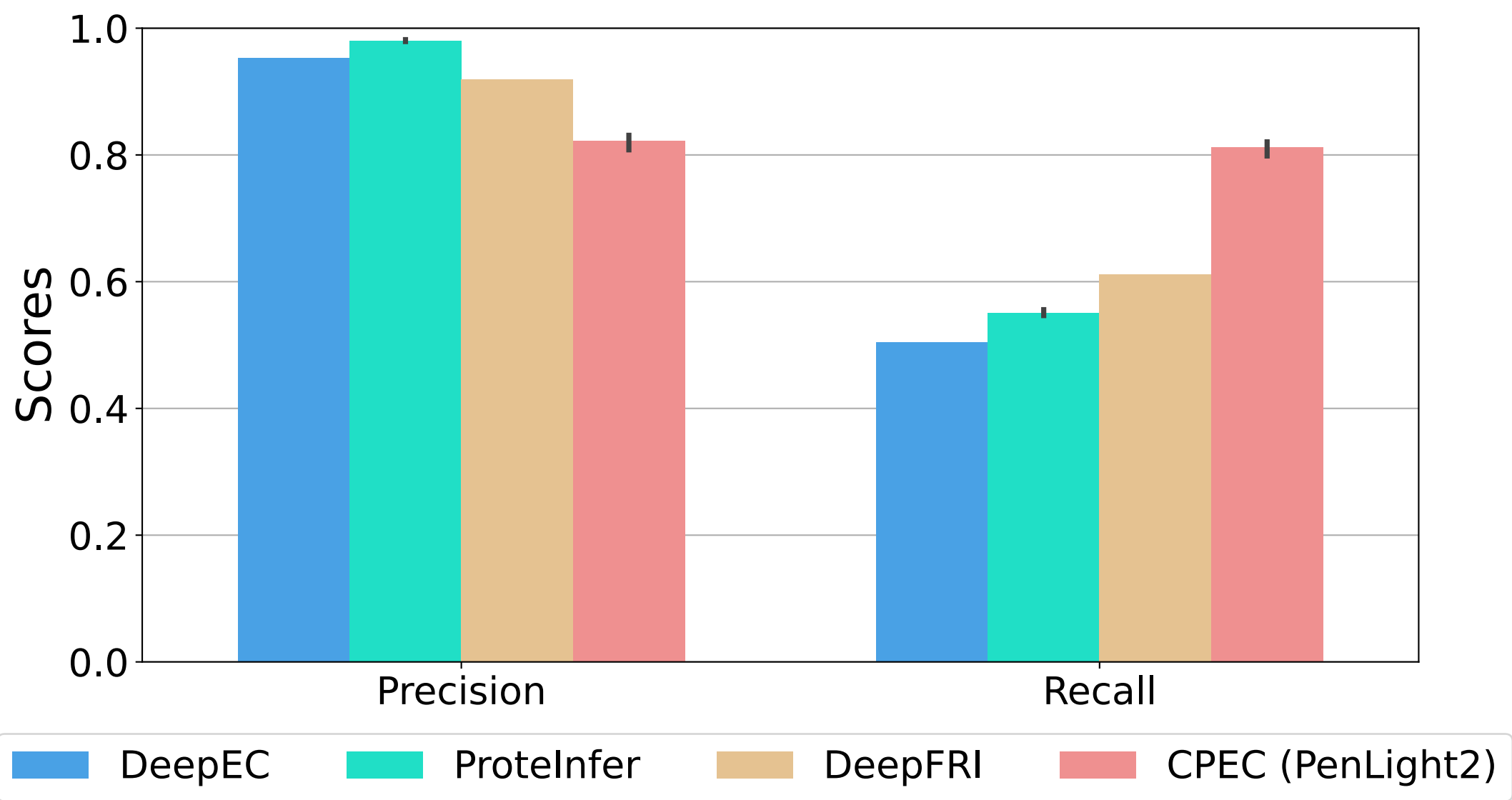

Supplement: S1 Fig — We evaluated DeepEC, ProteInfer, DeepFRI, and CPEC (PenLight2) for predicting the 4th level EC number, using sample-averaged precision and recall as the metrics. DeepEC and DeepFRI were evaluated using the only trained model provided in their repositories, whereas ProteInfer was assessed using 5 different trained models. DeepFRI was trained on the same dataset as PenLight2 while DeepEC and ProteInfer were trained by their respective datasets. PenLight2 was trained using 5 different seeds. (PDF) [file pcbi.1012135.s002.pdf]

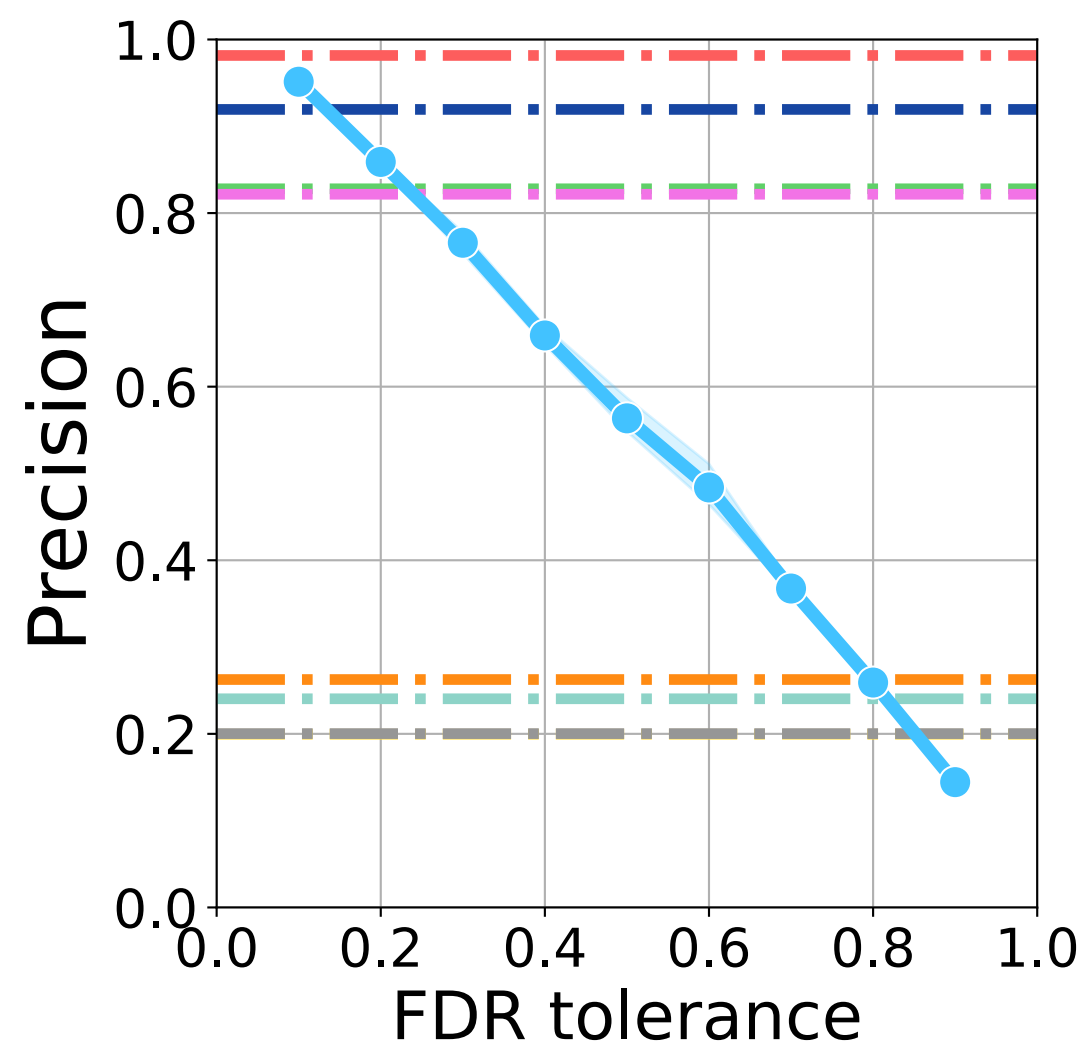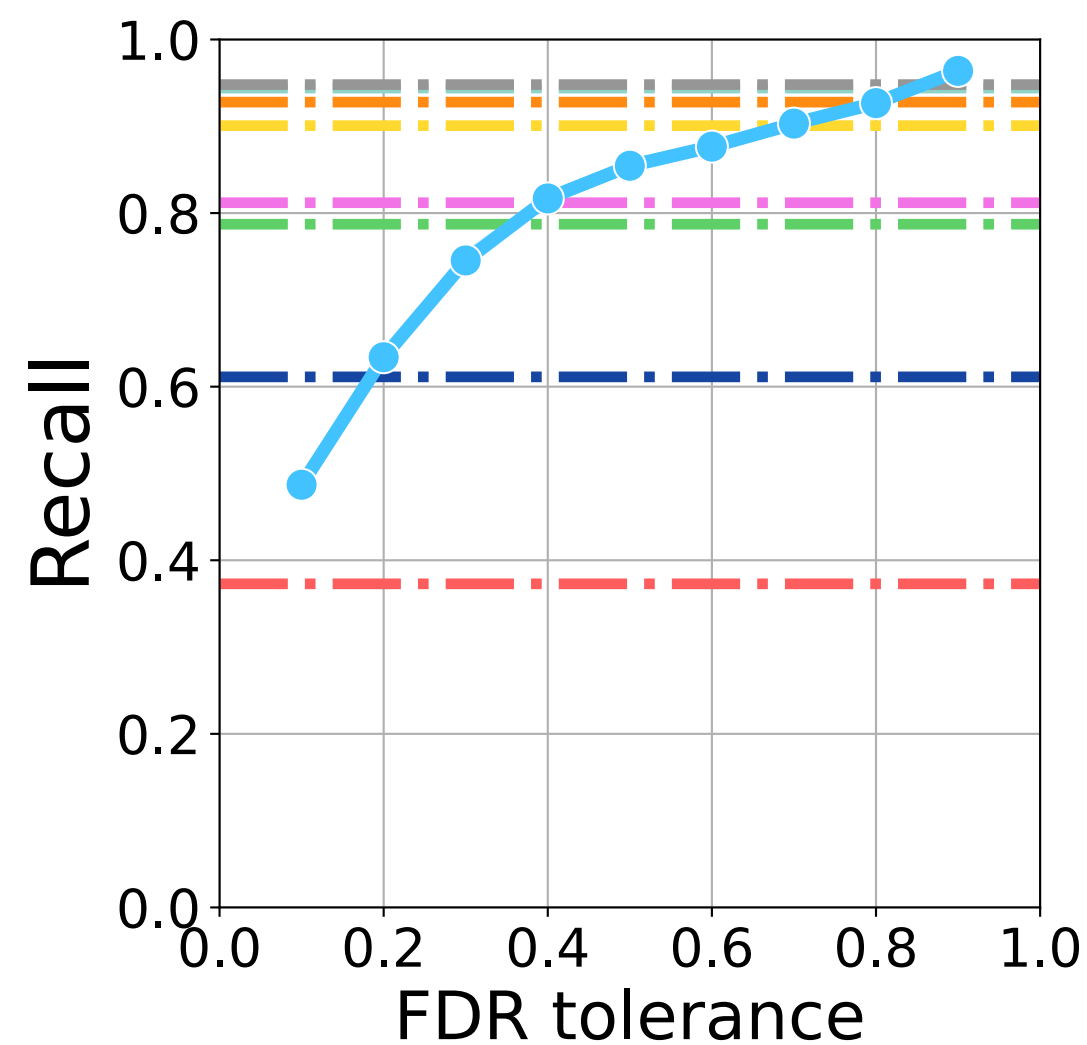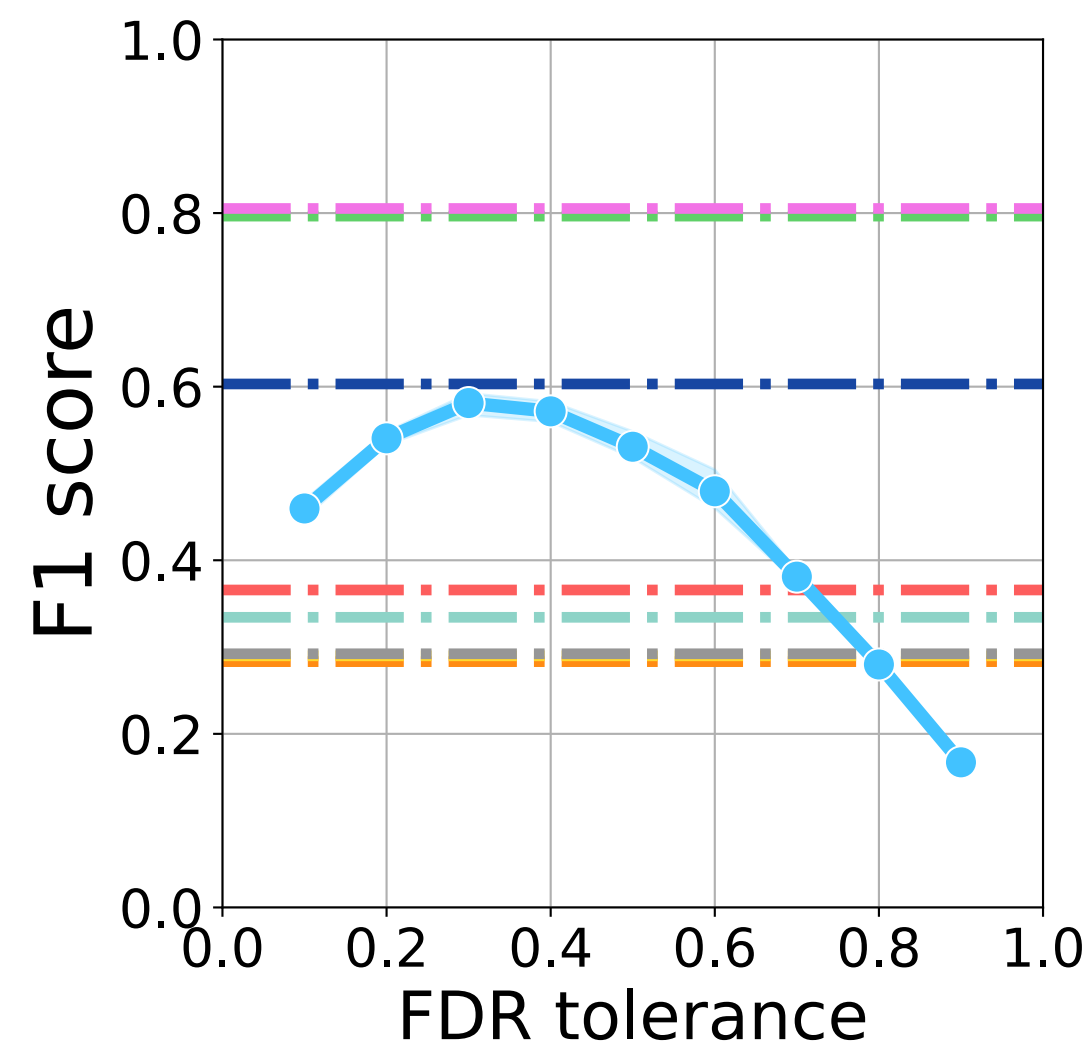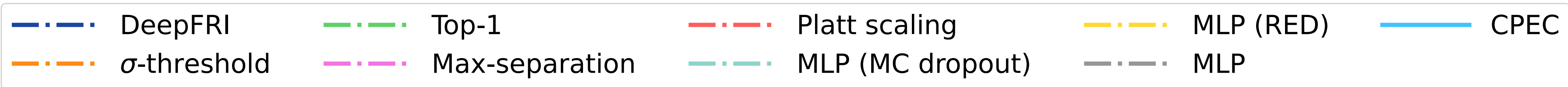

Supplement: S2 Fig — Platt scaling [34], RED [36], and Monte Carlo dropout [35] were further evaluated as thresholding strategies, in comparison to CPEC. Due to the requirements of the methods, RED and MC dropout were applied on top of an MLP model. The results of CPEC and all of the thresholding strategies were averaged over five different seeds. (PDF) [file pcbi.1012135.s003.pdf]

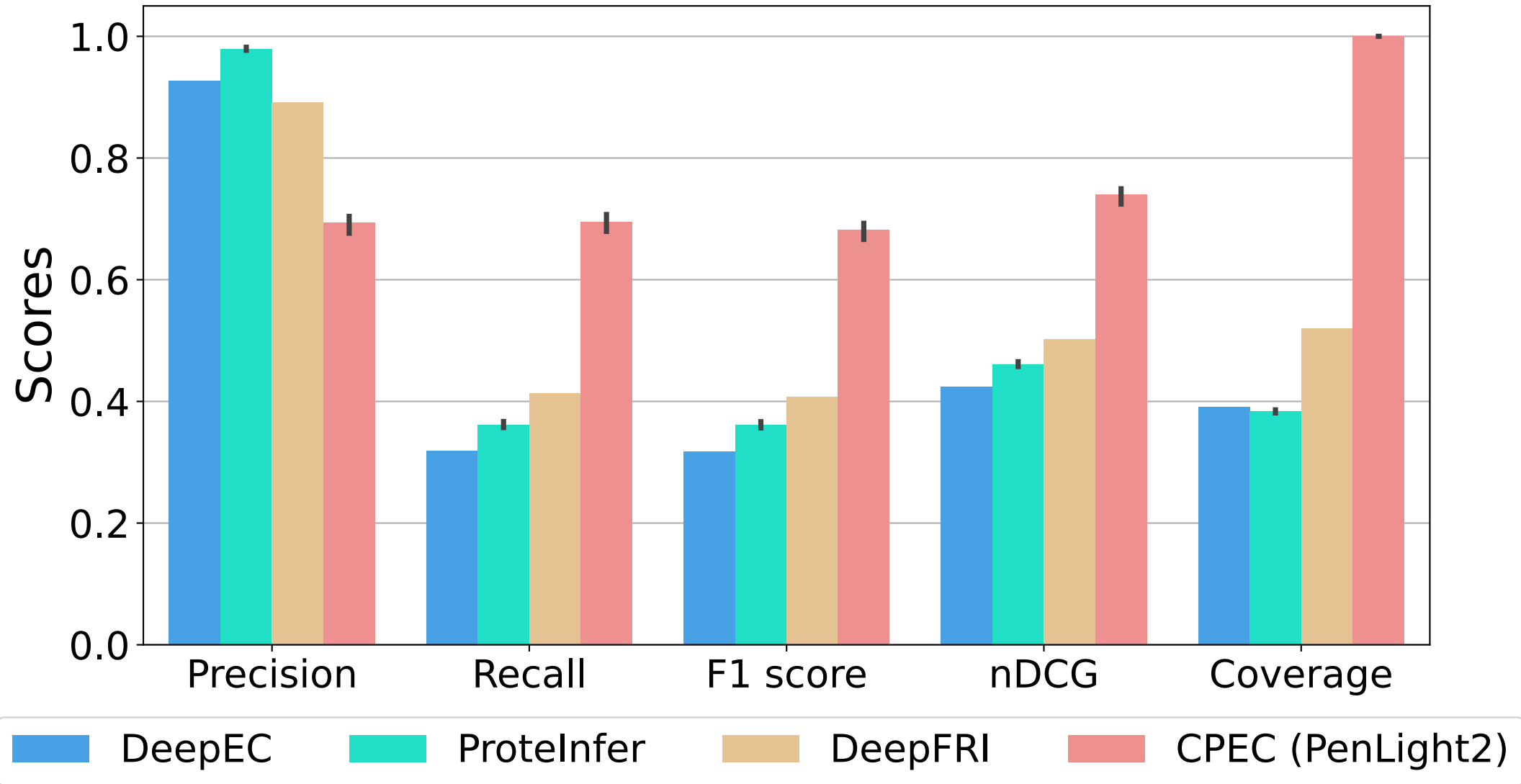

Supplement: S3 Fig — We evaluated DeepEC, ProteInfer, DeepFRI, and CPEC (PenLight2) for predicting the 4th level EC number, using sample-averaged precision, recall, F1 score, nDCG, and coverage as the metrics. DeepEC and DeepFRI were evaluated using the only trained model provided in their repositories, whereas ProteInfer was assessed using 5 different trained models. DeepFRI was trained on the same dataset as PenLight2 while DeepEC and ProteInfer were trained by their respective datasets. PenLight2 was trained using 5 different seeds. (PDF) [file pcbi.1012135.s004.pdf]

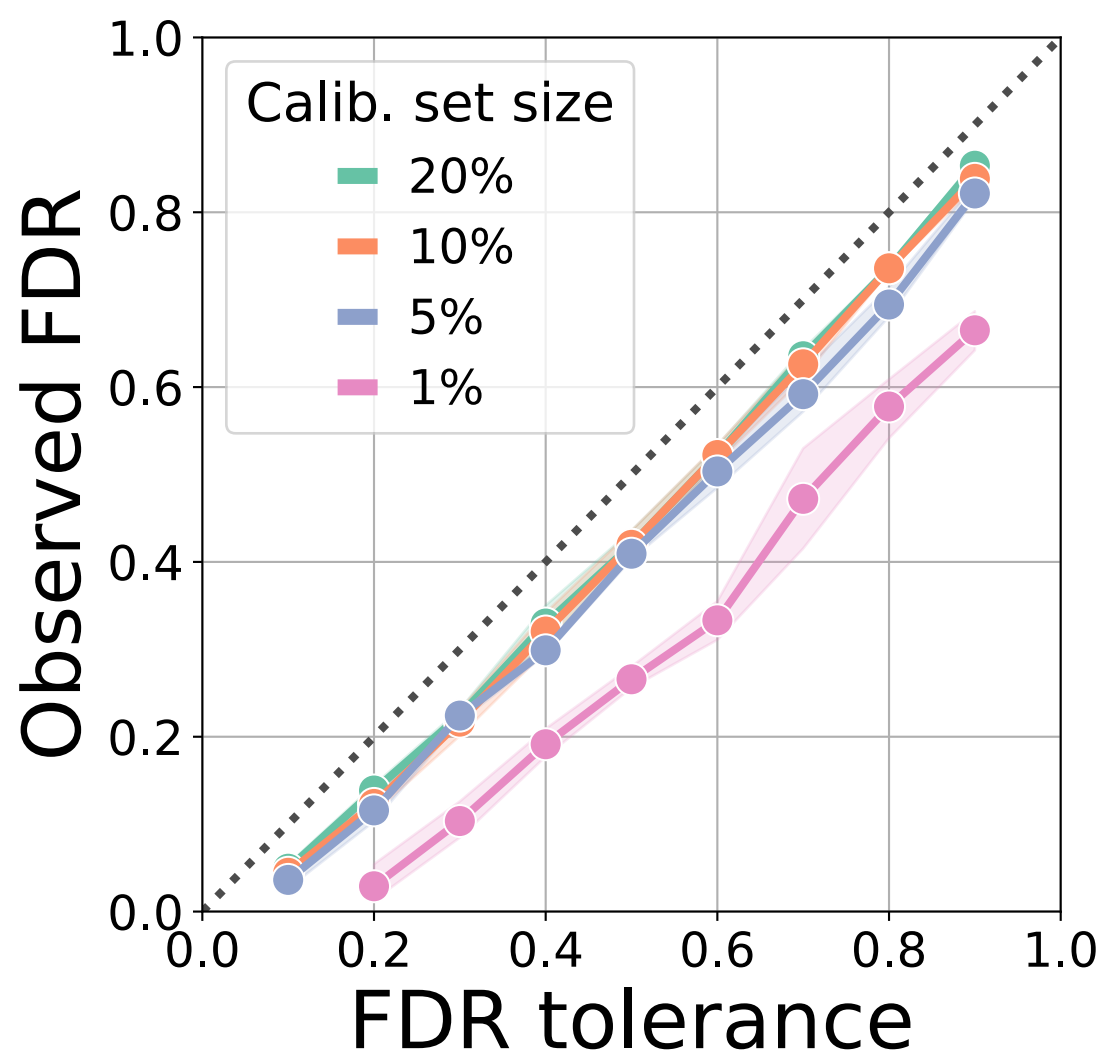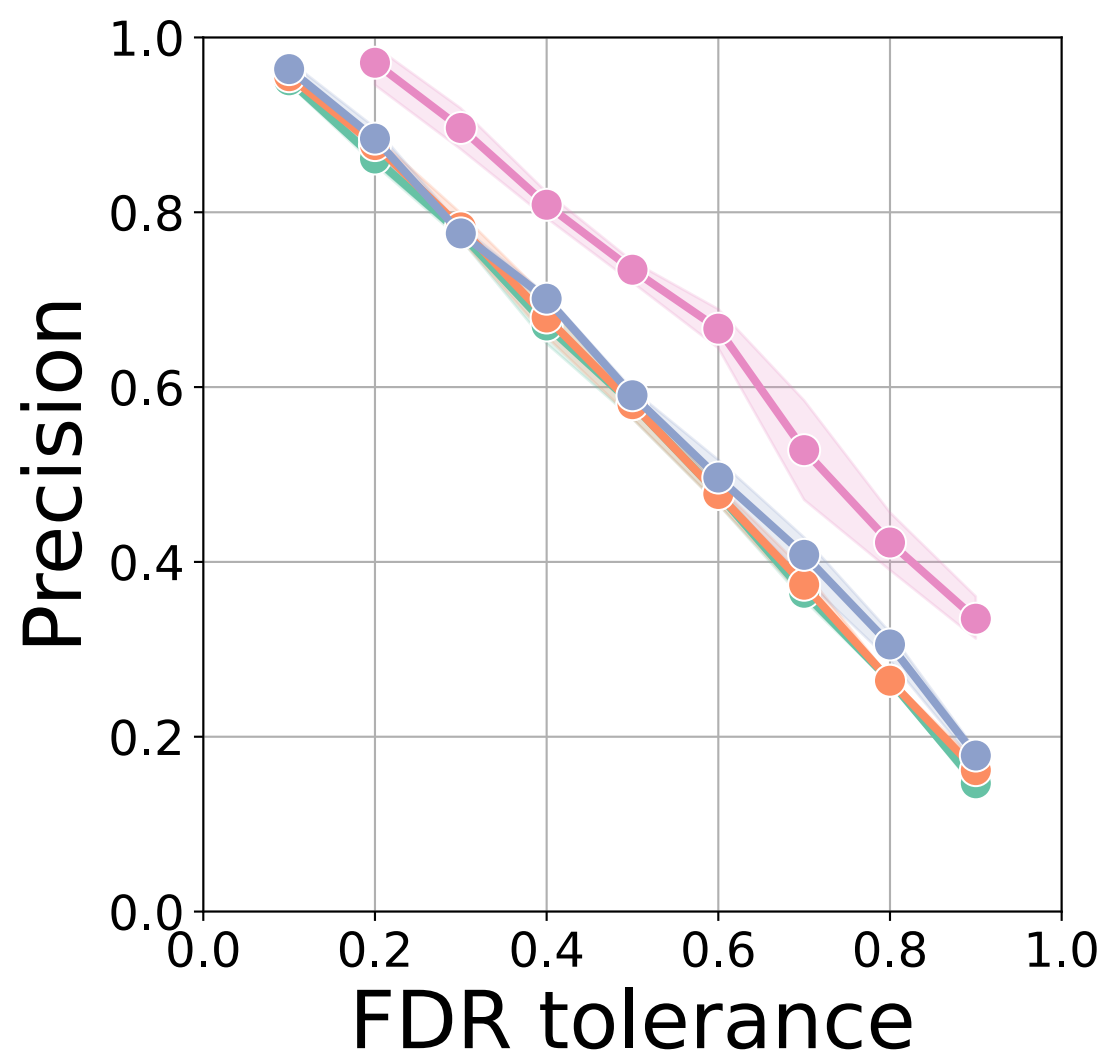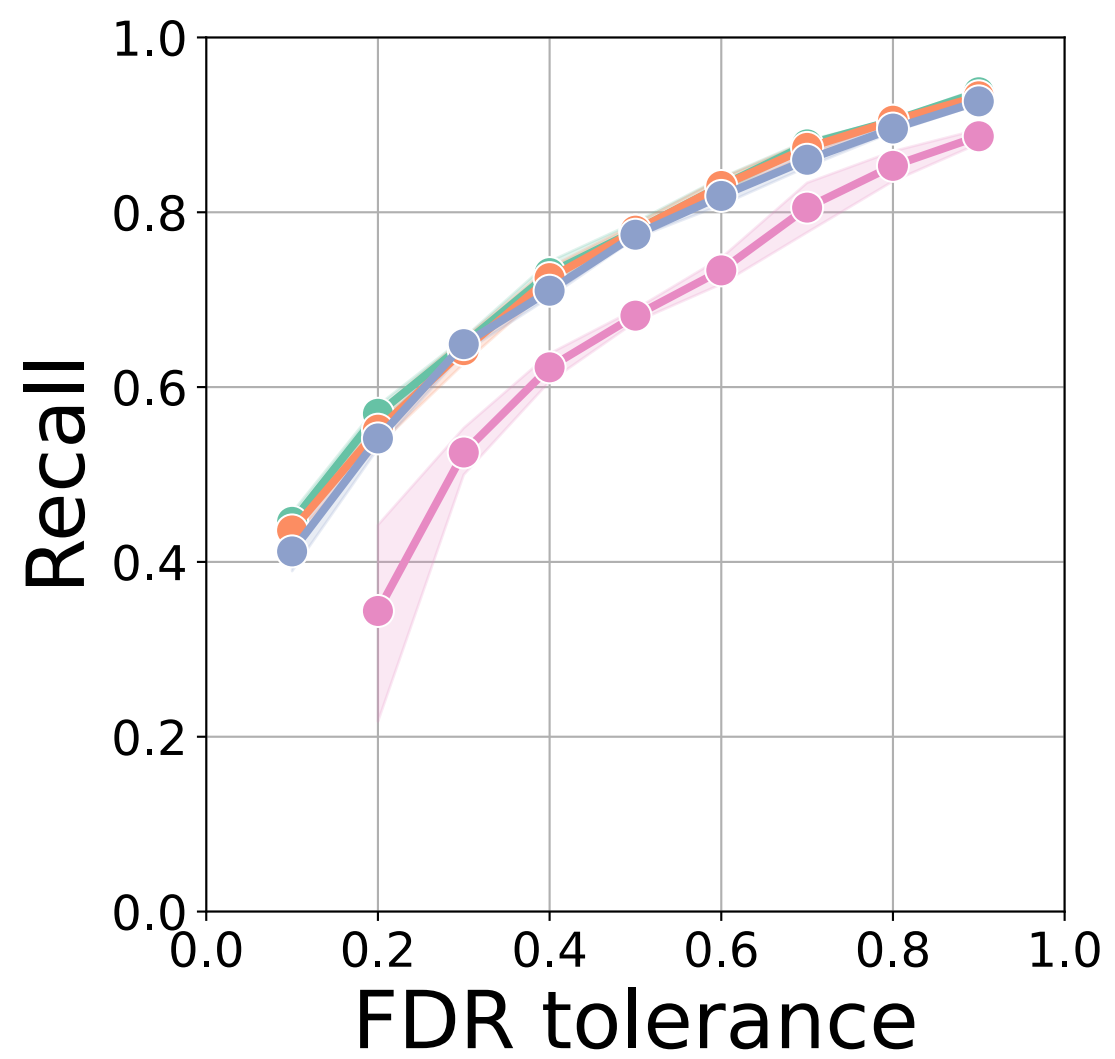

Supplement: S4 Fig — The performances of CPEC’s FDR control were evaluated using calibration sets with various sizes (abbrev: calib. set size): 20%, 10%, 5%, and 1% of the total number of the training data. The same training data was used across all calibration set sizes to ensure consistency in the comparison. The black dotted line in the first panel represents the theoretical upper bound of FDR over test proteins. The results were averaged over five different seeds. (PDF) [file pcbi.1012135.s005.pdf]

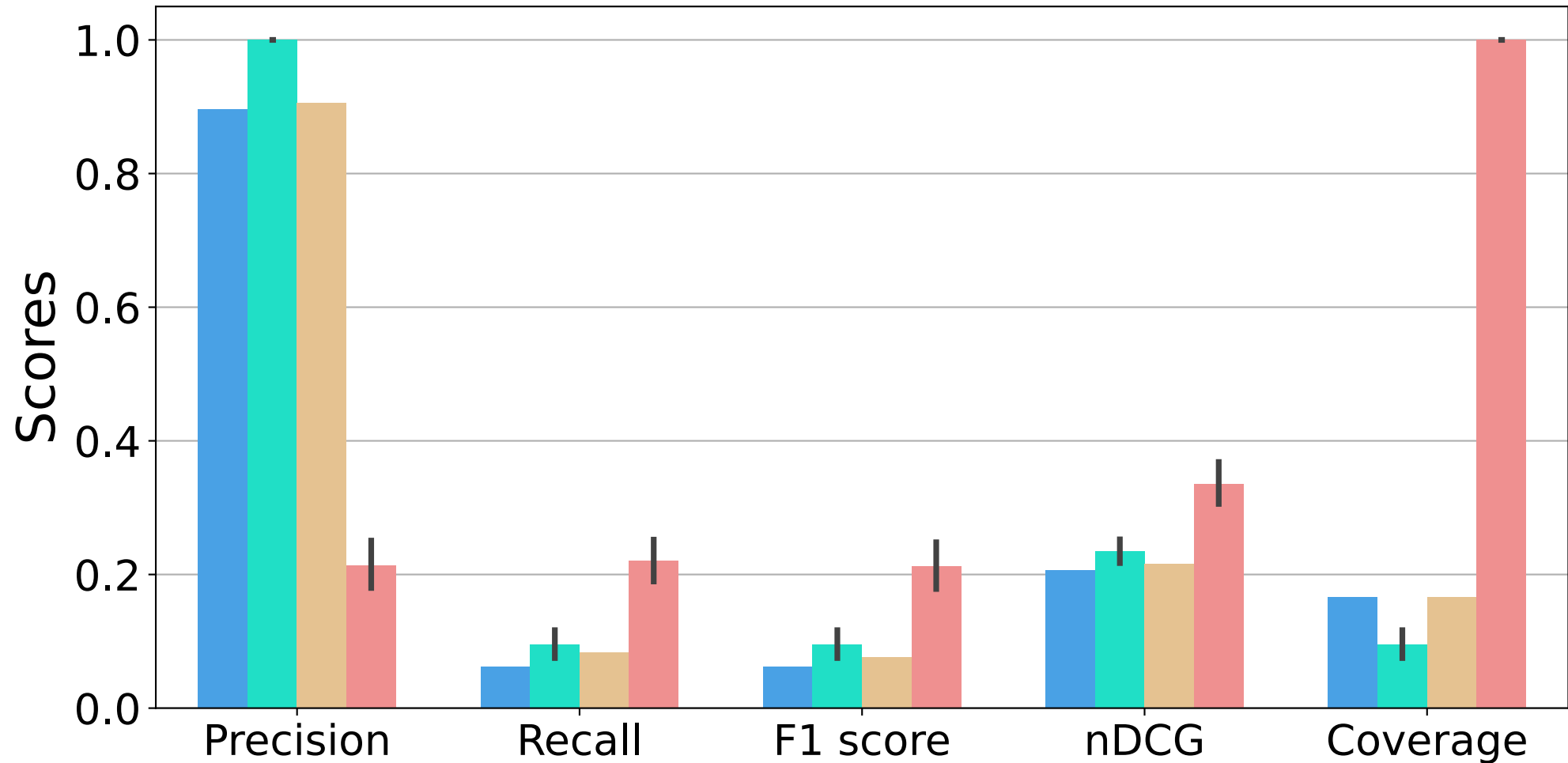

DeepEC   ProteInfer   DeepFRI   CPEC (PenLight2)

Supplement: S5 Fig — CPEC and three baseline methods (DeepEC, ProteInfer, and DeepFRI) were evaluated for predicting the 4th level EC number, using sample-averaged precision, recall, F1 score, nDCG, and coverage as the metrics. DeepEC and DeepFRI were evaluated using the only trained model provided in their repositories, whereas ProteInfer was assessed using 5 different trained models. DeepFRI was trained on the same dataset as CPEC, while DeepEC and ProteInfer were trained using their respective datasets. Training proteins not labeled in the CATH database were only removed from the training dataset of CPEC but not from the baseline methods’ training sets, which gave potential advantages to baseline methods. CPEC was trained using 5 different seeds. (PDF) [file pcbi.1012135.s006.pdf]

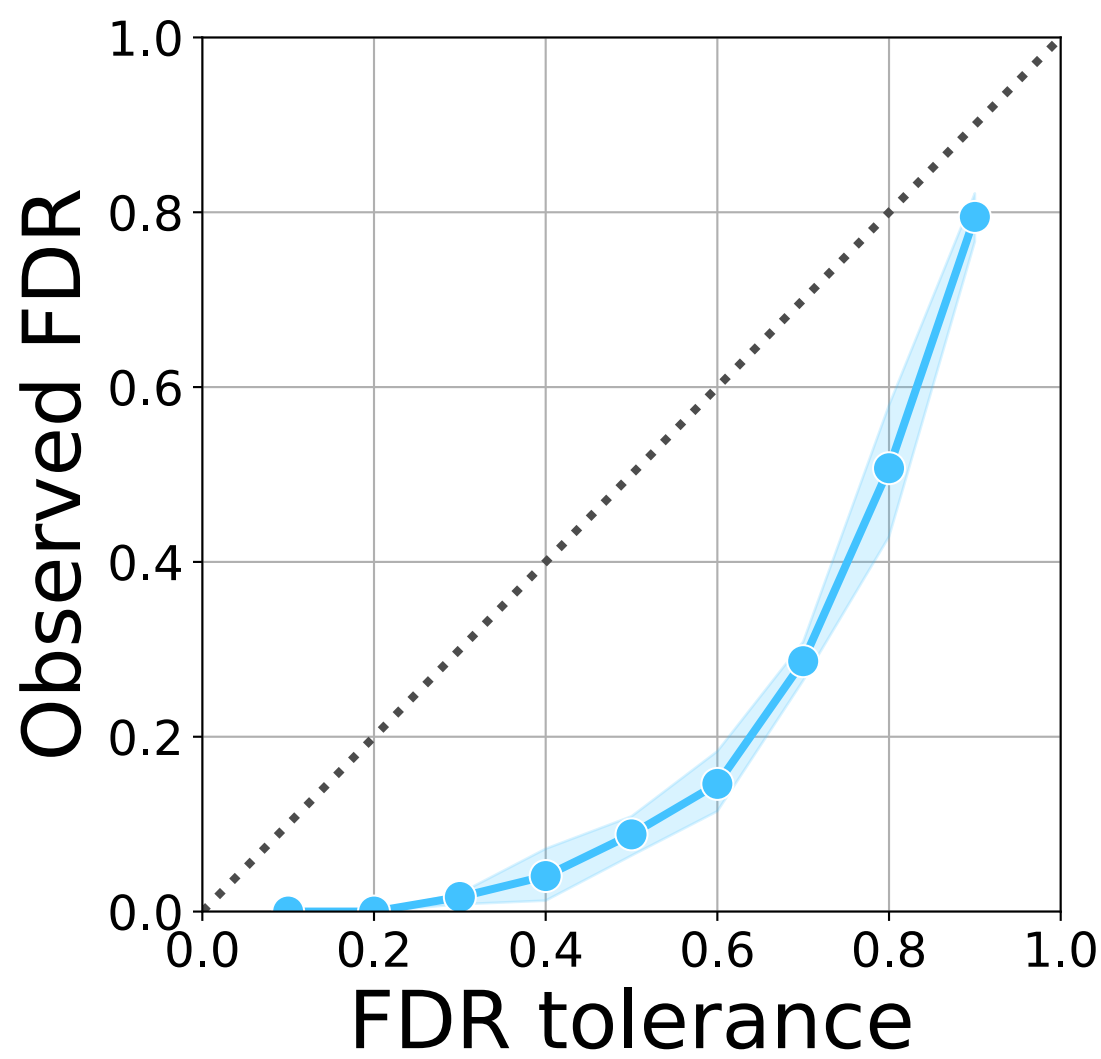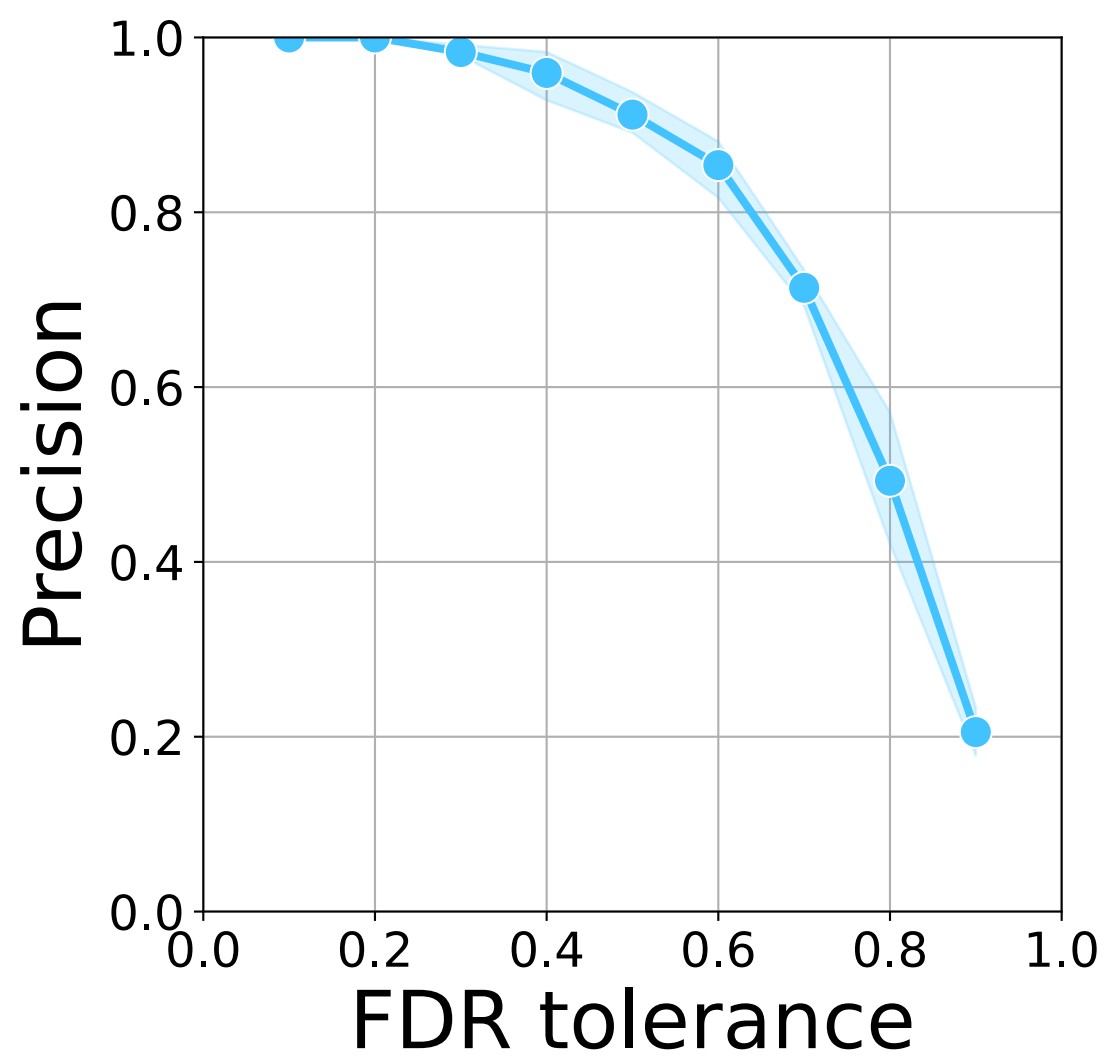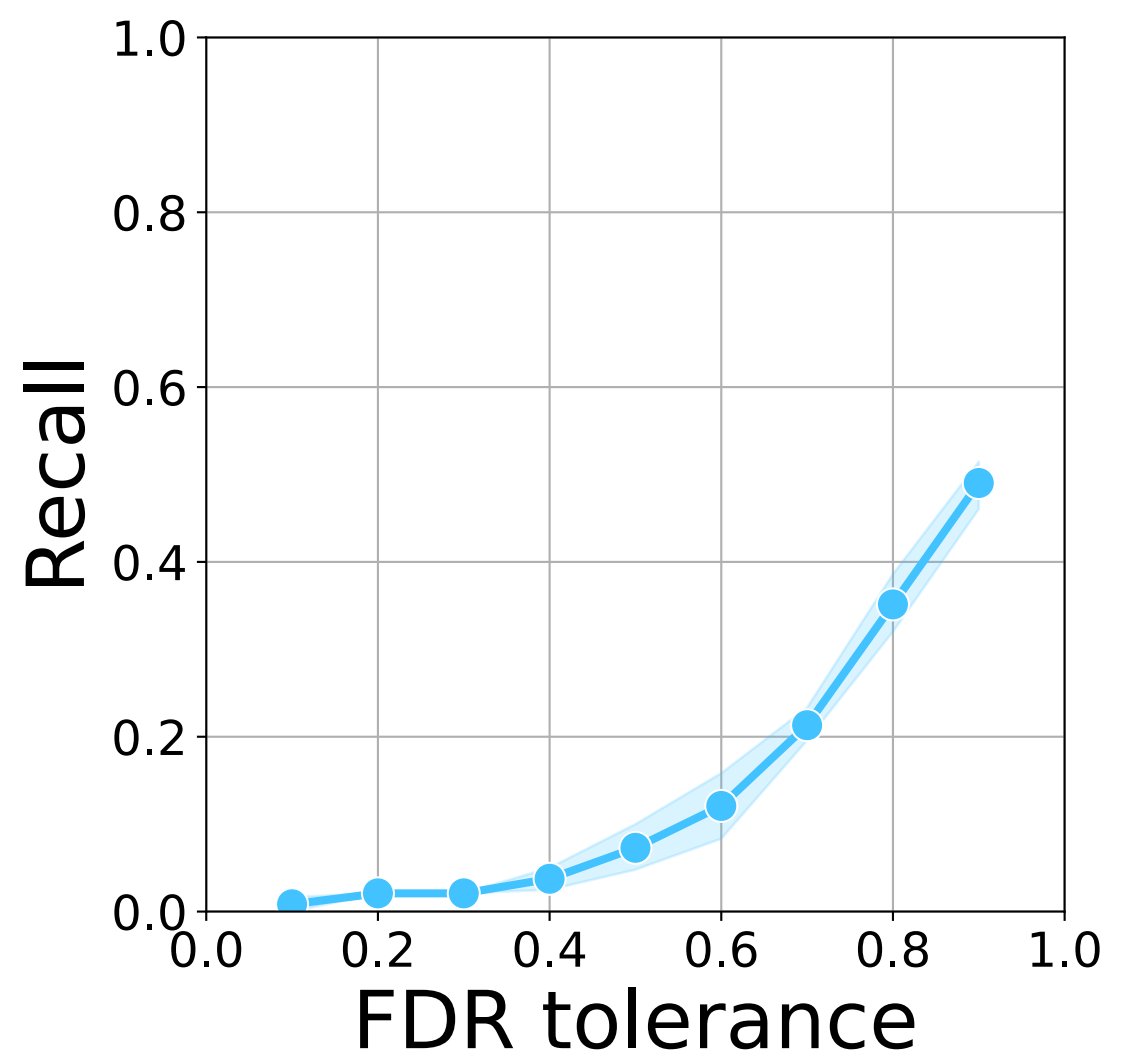

Supplement: S6 Fig — The FDR control of CPEC was evaluated on a more challenging data split: no training and test proteins belong to the same CATH [37] superfamily. Training proteins not labeled in the CATH database were only removed from the training dataset of CPEC but not from the baseline methods’ training sets, which gave potential advantages to baseline methods. The results were averaged over five different seeds. (PDF) [file pcbi.1012135.s007.pdf]

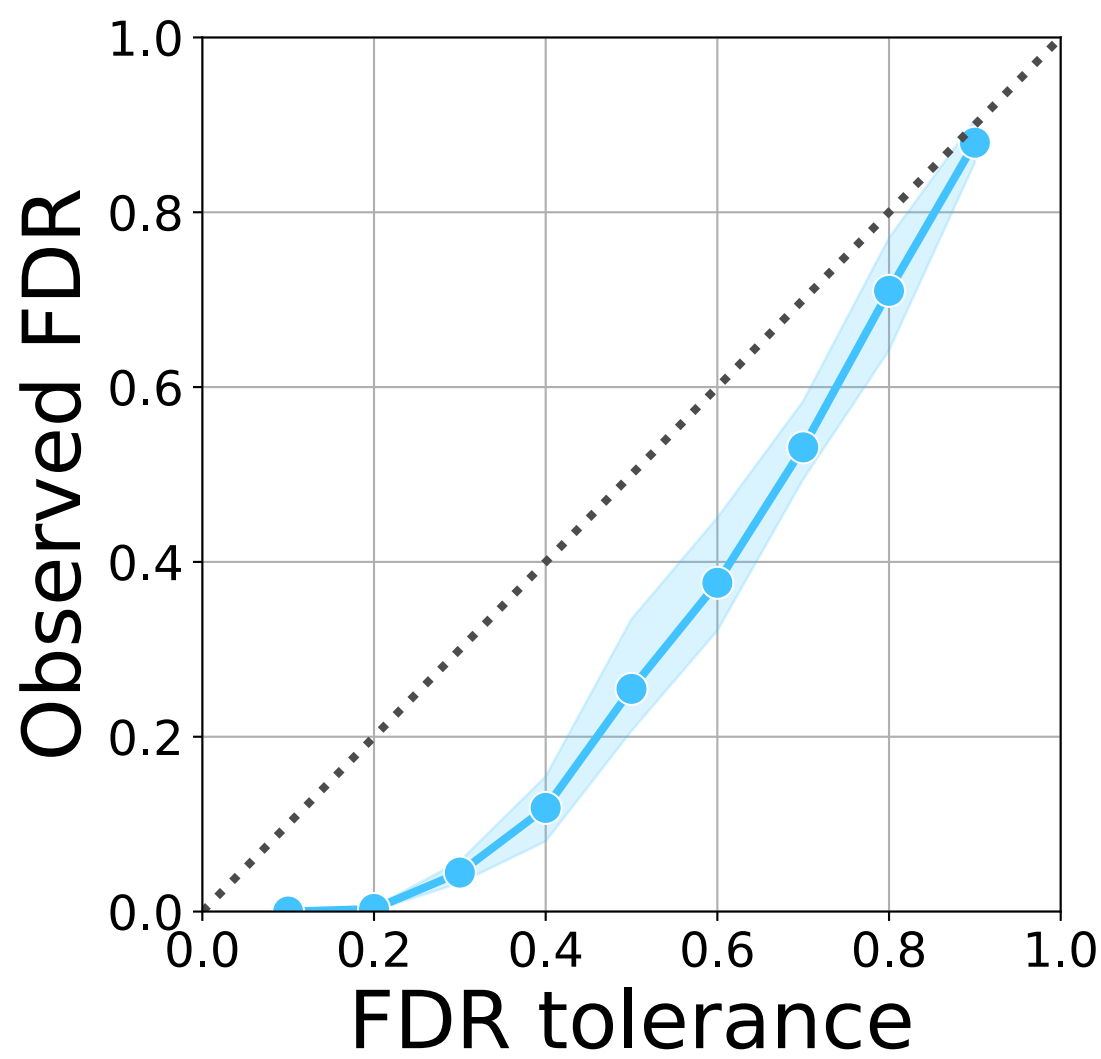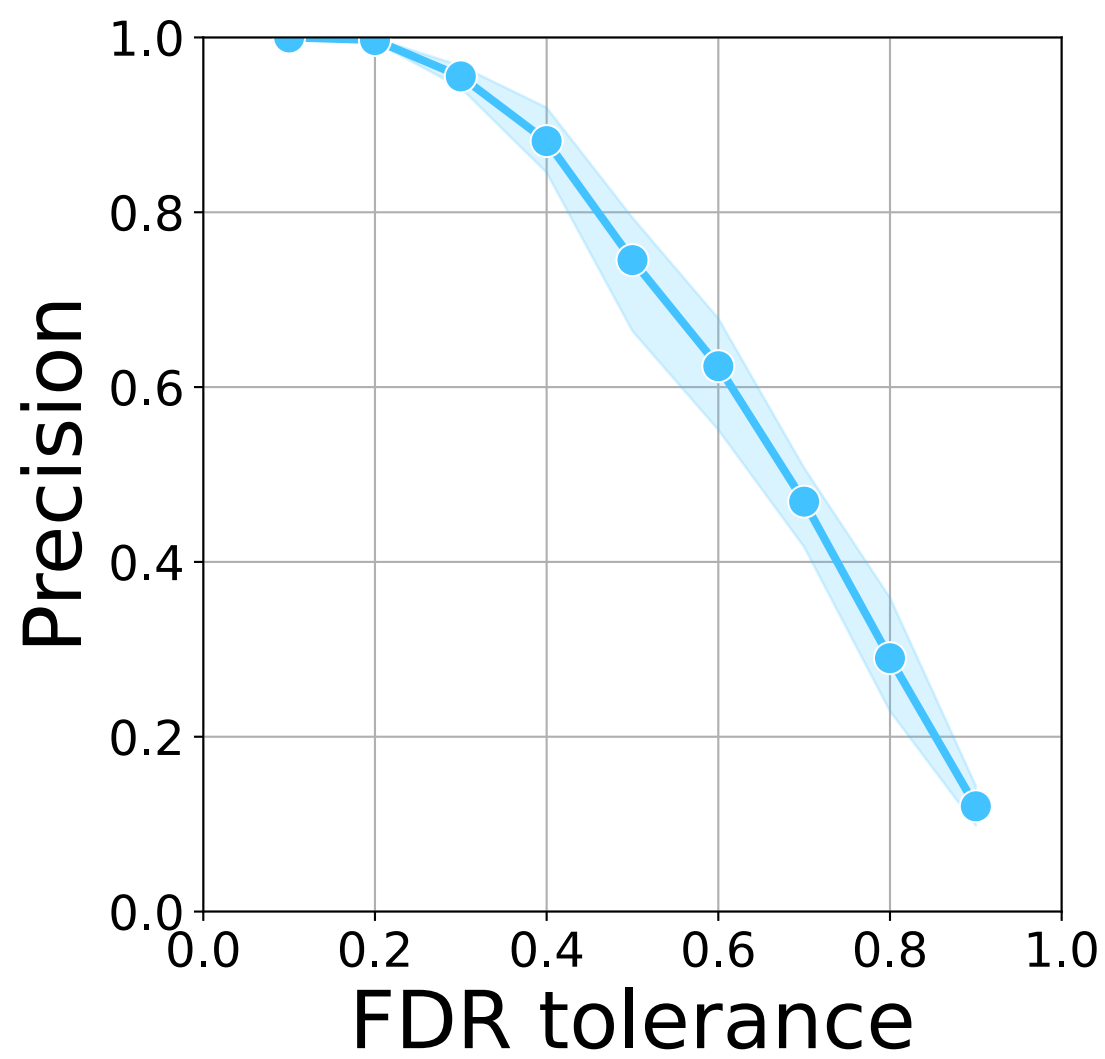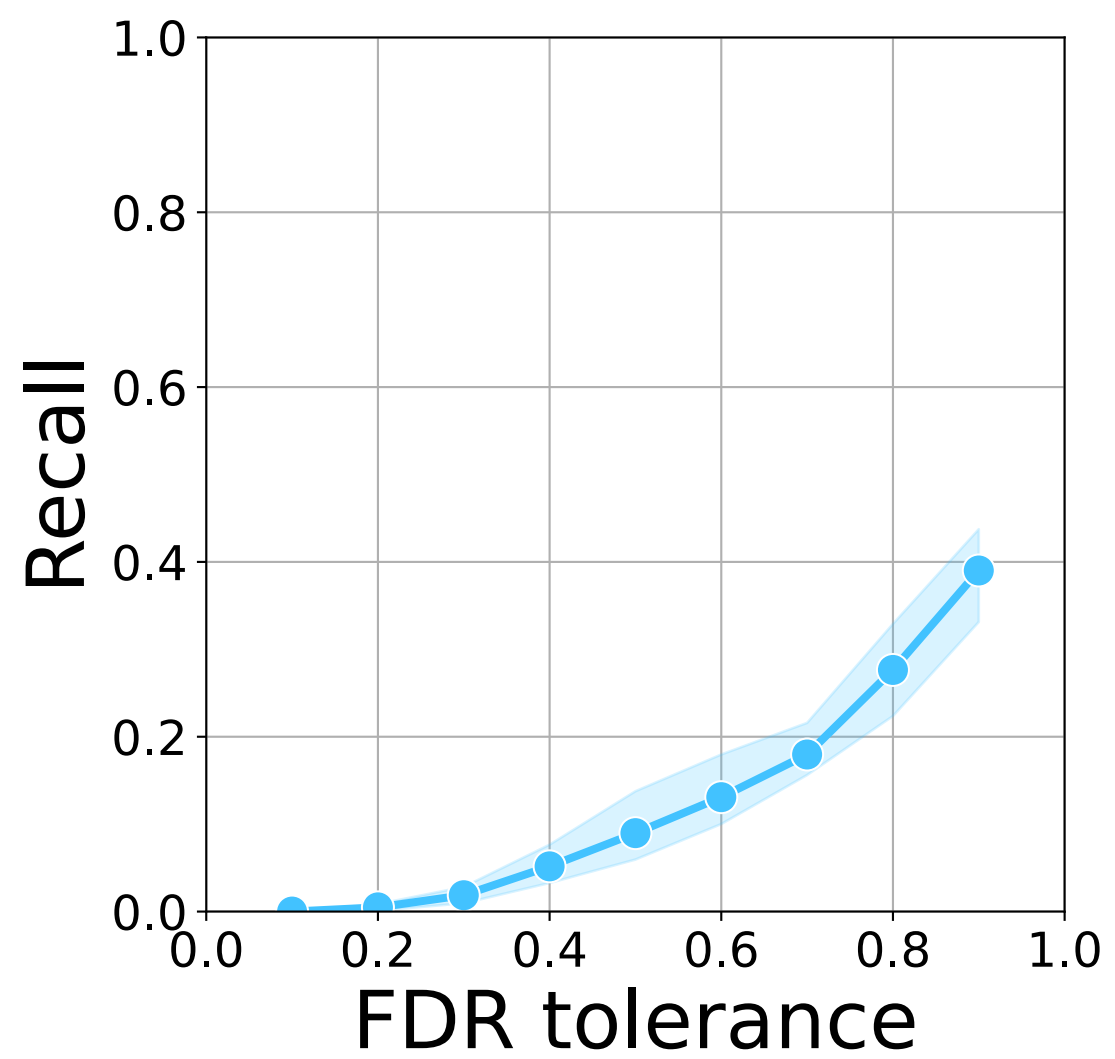

Supplement: S7 Fig — The FDR control of CPEC was evaluated on a more challenging data split: no training and test proteins belong to the same CATH [37] superfamily. A total number of 200 test proteins were sampled from the test set, and proteins that belong to the same superfamilies as the sampled test proteins were removed from the training set of CPEC. Training proteins not labeled in the CATH database were only removed from the training dataset of CPEC but not from the baseline methods’ training sets, which gave potential advantages to baseline methods. The results were averaged over five different seeds. (PDF) [file pcbi.1012135.s008.pdf]

# Precision-recall curve

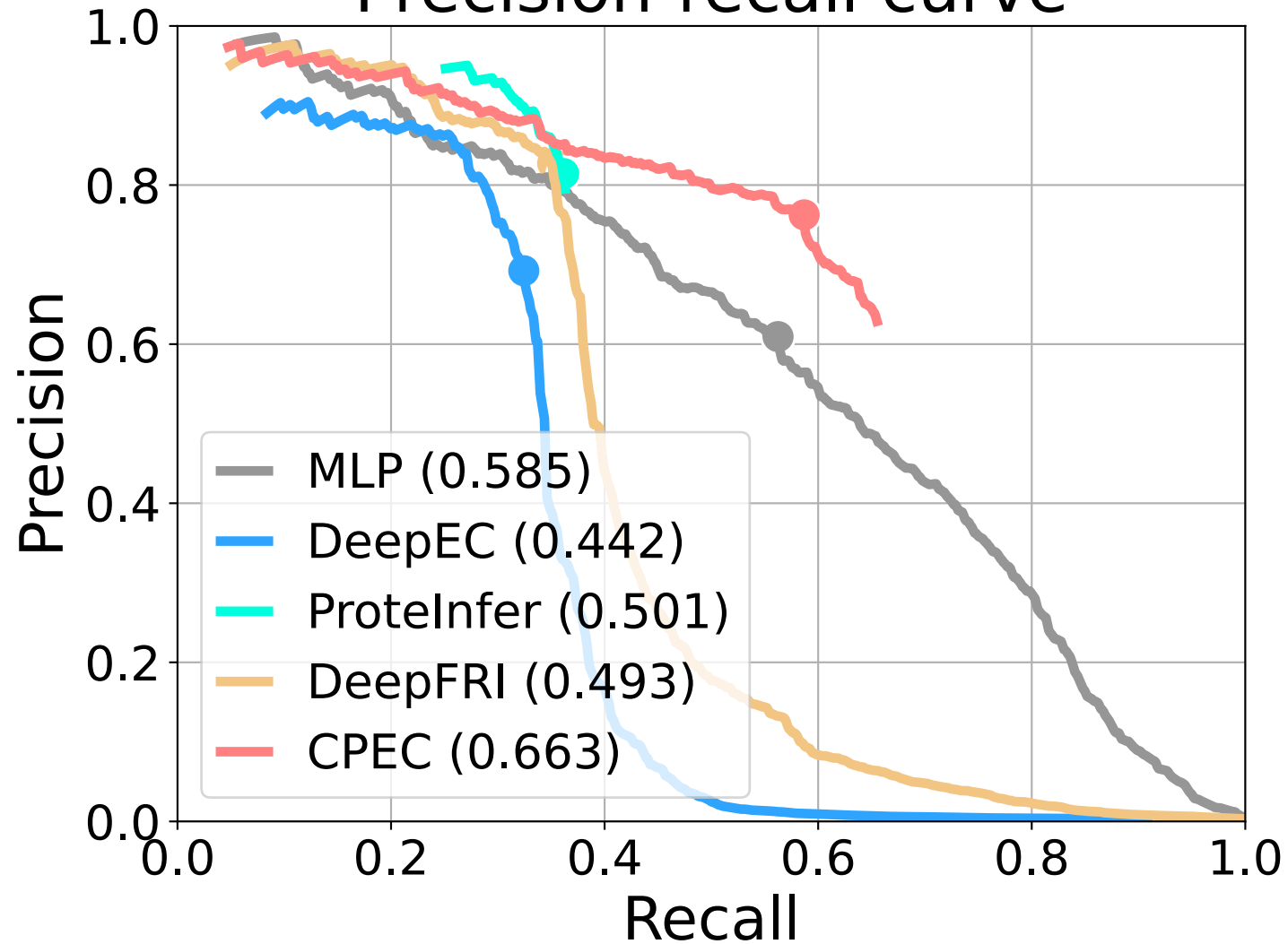

Supplement: S8 Fig — CPEC (PenLight2) and four baseline methods (including a baseline MLP model that takes the ESM-1b protein embeddings as the input) were evaluated for predicting the 4th-level EC number on more challenging test proteins with [0, 30%) sequence identities to the training proteins and the micro-averaged precision-recall curves were drawn. For each curve, the point with the maximum F1 score (Fmax) was labeled. (PDF) [file pcbi.1012135.s009.pdf]

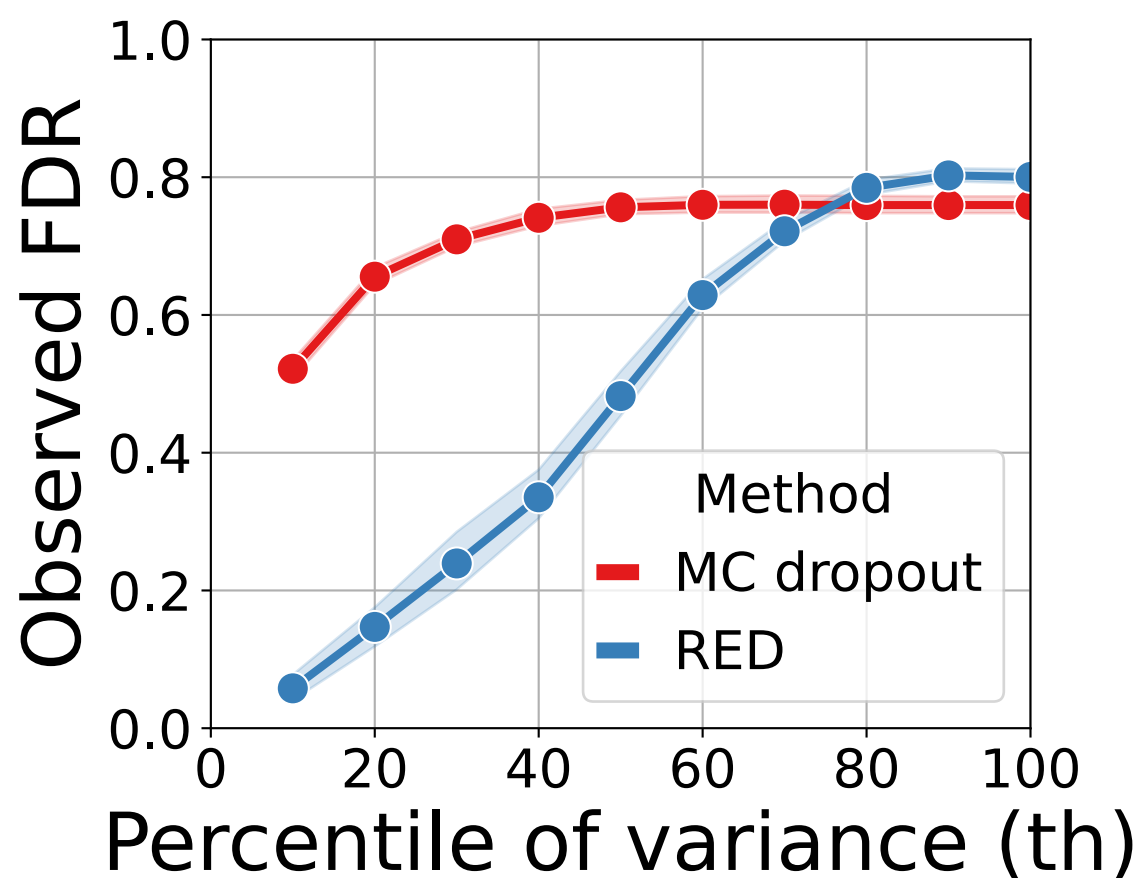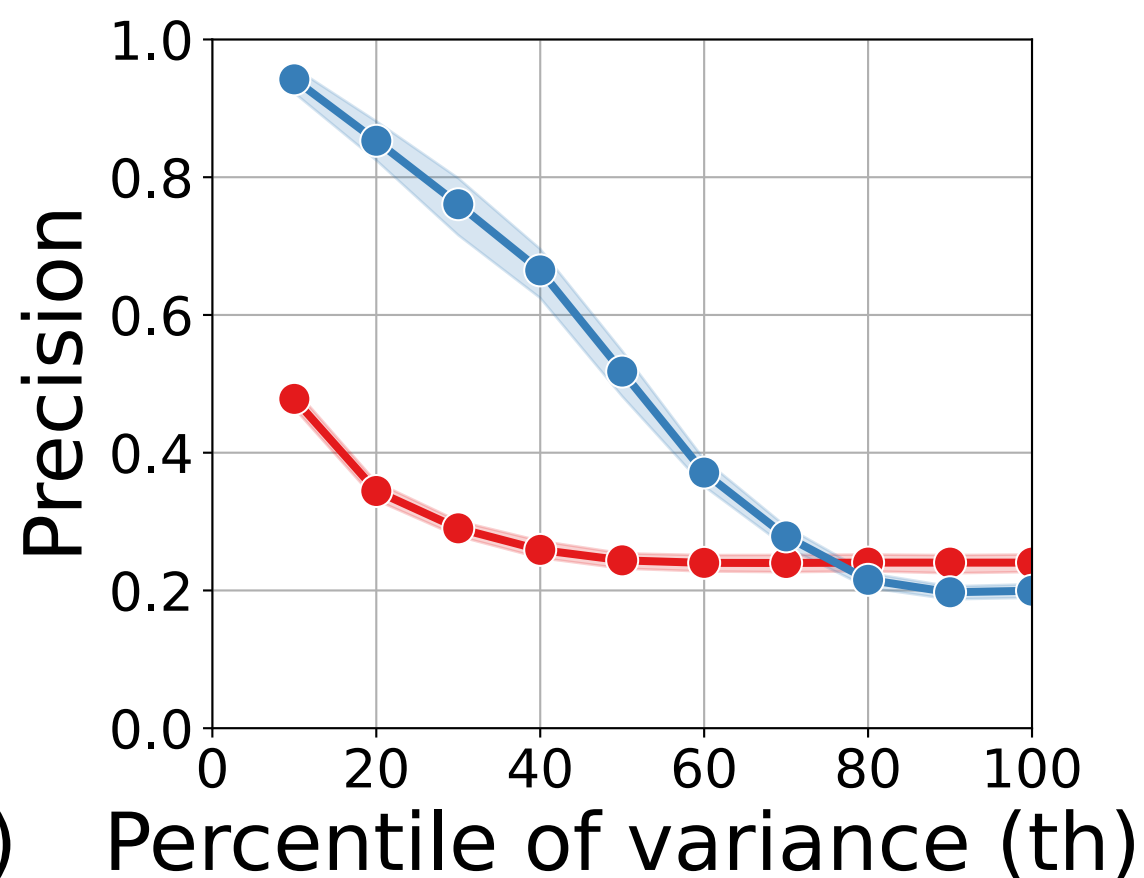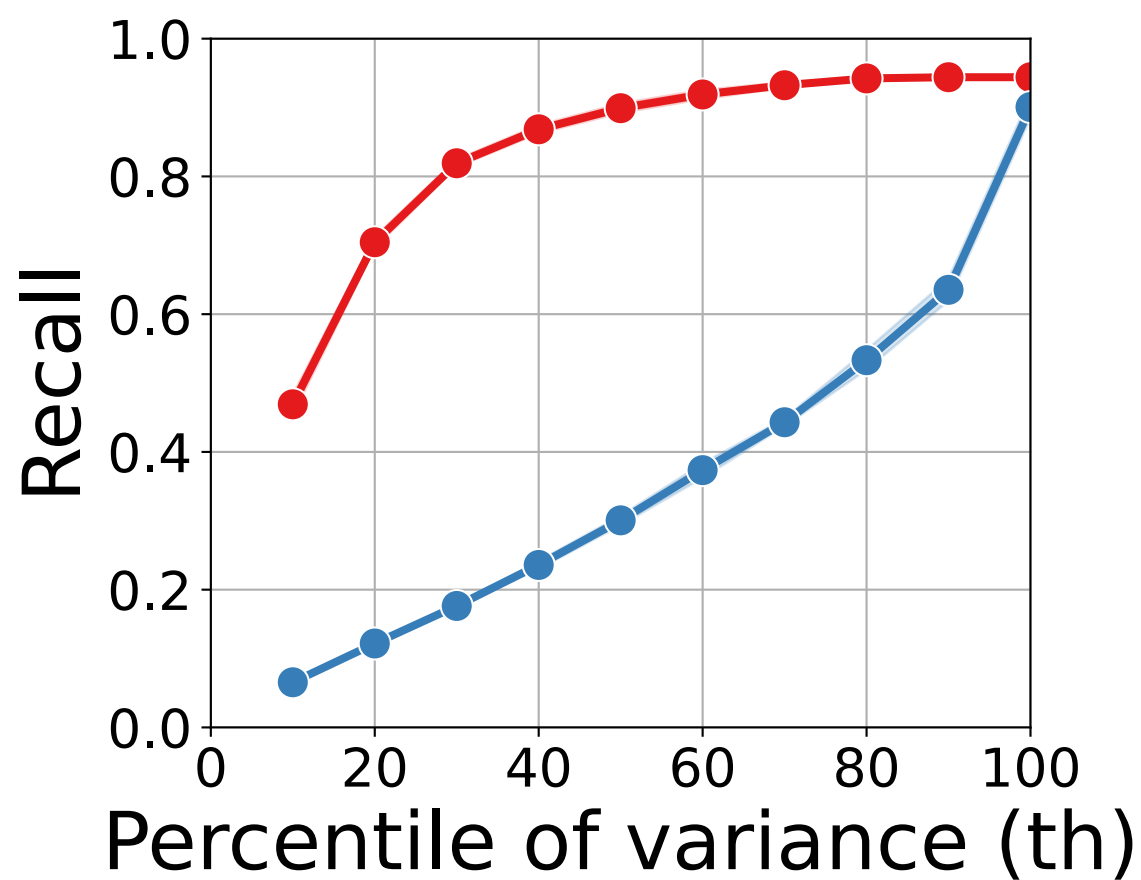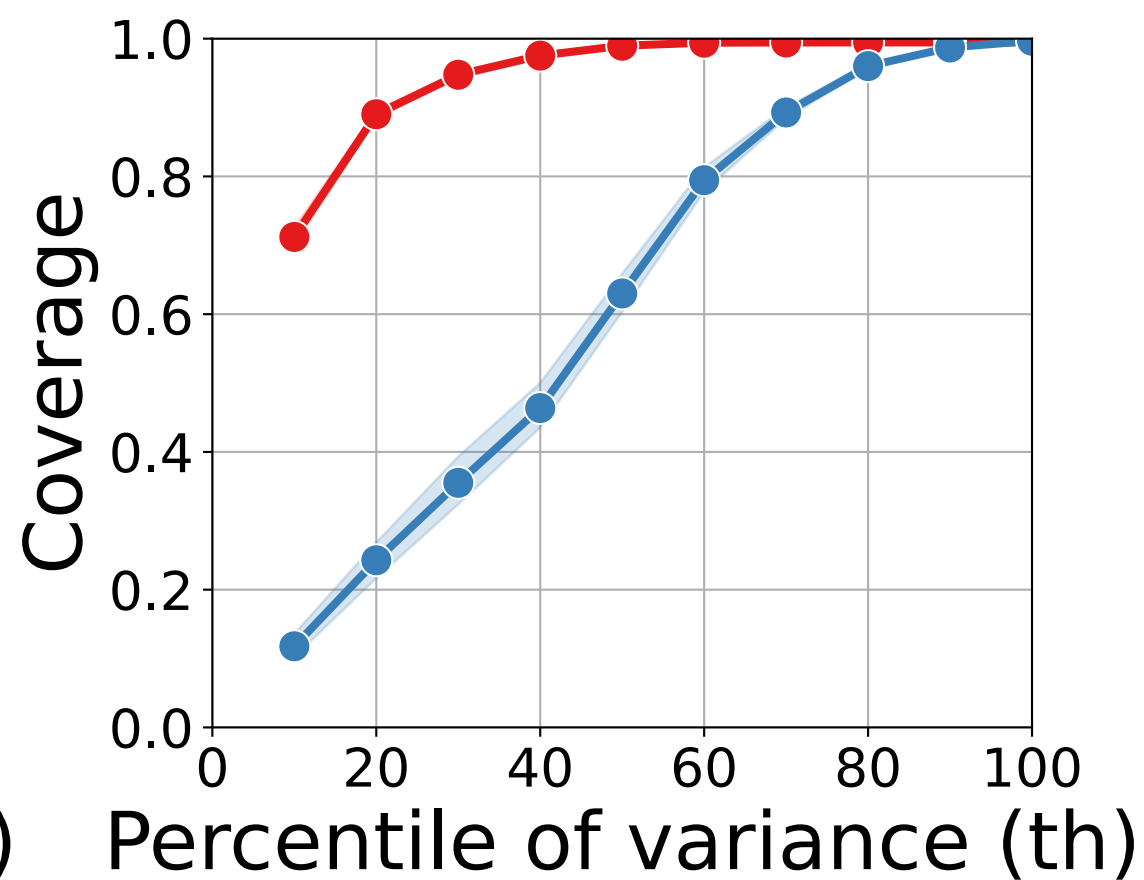

Supplement: S9 Fig — Two point-uncertainty prediction methods (Monte Carlo dropout (MC dropout) [35] and RED [36]) were evaluated in terms of uncertainty quantification. To make a fair comparison, a multi-layer perception taking ESM-1b protein embedding as the input was selected as the base ML model. The percentiles of the prediction variance (10th, 20th, 30th,…, and 100th percentiles) on the test set were used as the cutoffs. Predictions with variances larger than the cutoff were dropped. Observed false discovery rate (FDR), precision, recall, and coverage were used as metrics. The results were averaged over five different seeds. (PDF) [file pcbi.1012135.s010.pdf]
